# *In vitro* biological evaluation of a novel folic acid-targeted receptor quantum dot−*β*−cyclodextrin carrier for C−2028 unsymmetrical bisacridine in the treatment of human lung and prostate cancers

**DOI:** 10.1007/s43440-024-00606-4

**Published:** 2024-06-18

**Authors:** Joanna Pilch, Agnieszka Potęga, Patrycja Kowalik, Agata Kowalczyk, Piotr Bujak, Artur Kasprzak, Ewa Paluszkiewicz, Anna Maria Nowicka

**Affiliations:** 1 Faculty of Chemistry, Gdańsk University of Technology, Narutowicza 11/12 Str., Gdańsk, 80-233 Poland; 2 Institute of Physical Chemistry, Polish Academy of Science, Warsaw, Poland; 3 Faculty of Chemistry, University of Warsaw, Warsaw, Poland; 4 Faculty of Chemistry, Warsaw University of Technology, Warsaw, Poland

**Keywords:** Unsymmetrical bisacridine, Folic acid, Targeted cancer therapy, Drug delivery nanosystem, Biological response

## Abstract

**Background:**

Traditional small-molecule chemotherapeutics usually do not distinguish tumors from healthy tissues. However, nanotechnology creates nanocarriers that selectively deliver drugs to their site of action. This work is the next step in the development of the quantum dot−*β*−cyclodextrin−folic acid (QD−*β*−CD−FA) platform for targeted and selected delivery of C−2028 unsymmetrical bisacridine in cancer therapy.

**Methods:**

Herein, we report an initial biological evaluation (using flow cytometry and light microscopy) as well as cell migration analysis of QD−*β*−CD(C−2028)−FA nanoconjugate and its components in the selected human lung and prostate cancer cells, as well as against their respective normal cells.

**Results:**

C−2028 compound induced apoptosis, which was much stronger in cancer cells compared to normal cells. Conjugation of C−2028 with QD_green_ increased cellular senescence, while the introduction of FA to the conjugate significantly decreased this process. C−2028 nanoencapsulation also reduced cell migration. Importantly, QD_green_ and QD_green_−*β*−CD−FA themselves did not induce any toxic responses in studied cells.

**Conclusions:**

In conclusion, the results demonstrate the high potential of a novel folic acid-targeted receptor quantum dot−*β*−cyclodextrin carrier (QD_green_−*β*−CD−FA) for drug delivery in cancer treatment. Nanoplatforms increased the amount of delivered compounds and demonstrated high suitability.

**Supplementary Information:**

The online version contains supplementary material available at 10.1007/s43440-024-00606-4.

## Introduction

Cancers or malignant neoplasms are still a leading cause of morbidity and mortality worldwide [1, 2]. The conventional strategies in cancer treatment involve surgical resection of the solid localized tumors, radiotherapy with X-rays, and chemotherapy used as a single treatment or in combination [3–6]. Although all of them are the most recommended, they are often not enough to provide an effective cure and/or protection from this devastating disease. Chemotherapy, which generally uses small-molecule compounds to kill fast-growing cancer cells and/or delay their growth, is one of the most common methods of treatment for blood-related cancers and solid metastatic tumors [7]. However, the development of a potent anticancer drug is a highly complex and multi-factorial process that faces many difficulties. Drug low water solubility, rapid metabolic decomposition, and aggregation could reduce its bioavailability [8, 9]. An important factor that limits drug clinical application is its non-selective action, which consists of toxicity to all rapidly proliferating cells– both cancer and normal cells [6, 10, 11]. This may lead to some serious adverse effects (e.g., side effects and multidrug resistance) or even cause the patient’s death [12].

The urgent need to obtain a ‘smart’ drug that can distinguish tumors from normal tissues, so it is transported to exactly the right place of action, raises progress in nanotechnology [12]. In recent years, nanoscale technology has provided a wide spectrum of opportunities to design and develop multi-functional devices that can target, diagnose, and treat cancers with high efficiency [13]. One example of such innovative strategies is nanoparticle-based platforms for the targeted delivery and release of drugs, proteins, peptides, or nucleic acids [11, 14, 15]. Nanoparticles (NPs) present in these systems include but are not limited to micelles, liposomes, dendrimers, carbon nanotubes, magnetic NPs, and quantum dots (QDs) [16]. They are usually designed or chosen based on their size and characteristics according to the pathophysiology of the tumors. Compared to traditional anticancer drugs, NP-based drug carriers have specific advantages, such as increased stability and biocompatibility, enhanced permeability, and retention effect [17, 18]. The improvement of drug bioavailability in tumor tissue is achieved by exploiting the differences between the normal physiological environment and the tumor microenvironment (TME) [19, 20]. The unique properties of TME, which are closely related to the occurrence and development of tumors, include acidity, hypoxia, excessive reactive oxygen species, and high glutathione concentration as well as overexpression of specific enzymes and receptors. Hence, TME-stimuli-responsive systems, such as pH-responsive, hypoxia-responsive, redox-sensitive, or targeting molecule-conjugated nanocarriers, are gaining more attention [19–21]. Active targeting of neoplastic cells in the latter can be realized through direct interactions between ligands on the surface of NPs and receptors overexpressed on the surface of cancer cells [12]. In view of this, promising tumor targets are folic acid (FA) receptors (FRs) as their expression is often enhanced on the surface of 40% of solid tumors, but negligible in the majority of healthy tissues [22]. Folic acid-drug delivery systems can enter tumor cells through receptor-mediated endocytosis which allows internalized NPs to successfully release drug molecules [23]. In this way, it improves the drug’s therapeutic efficacy while protecting normal cells from cytotoxicity. Other docking sites for precise targeting of anticancer chemotherapeutics include receptors for transferrin (TfR) [24, 25], G-proteins (GPCRs) [26], or epidermal growth factor (EGFR) [27].

Considering the above, we previously constructed the quantum dots−*β*−cyclodextrin−folic acid (QD− *β*−CD−FA) vehicles as a platform for improved unsymmetrical bisacridine (UA) delivery and controllable drug release in various human cancer cells [28–31]. It was fully structurally and morphologically characterized [28, 30, 31]. The details can also be found in the Supplementary information. In this multi-component system, we applied quaternary QDs (Ag−In−Zn−S nanocrystals) decorated with *β*−CD which was the main skeleton for drug encapsulation. QDs represent a promising avenue for the design and engineering of versatile drug nanocarriers. A combination of unique physical, chemical, and optical properties enables the study of QD interactions with biological systems through real-time monitoring of NP biodistribution, intracellular uptake, controlled drug release, and long-term nanocarrier fate. Therefore, QDs have a significant impact on the further development of life sciences, including their applications in bio-imaging, diagnostics, and biosensing [32, 33]. CDs are amphiphilic cyclic oligosaccharides. Due to their unique internal cavity, CDs are regarded to be an efficient and accessible unit for the construction of drug delivery systems. They are characterized by high stability, good biocompatibility, low immunogenicity, negligible toxicity, and modifiability. In addition, CDs can circumvent the limitations of NPs, such as low encapsulation efficiency and drug loading [34]. FA was used as a self-navigating molecule to specifically bind to FRs. C−2028 was selected as a model compound from a novel class of patented anticancer drug candidates - UAs - developed in our research team. These acridine analogs, in non-conjugated form, exhibited high cytotoxic activity against a lot of tumor cells *in vitro* (e.g., human breast, prostate, pancreatic, colon, and lung cancers) as well as demonstrated high antitumor efficacy against several types of human cancer xenografts in nude mice, including pancreatic, colorectal, and lung cancers [35]. So far, we have shown that the application of QDs and FA successfully increased the cellular uptake of the studied C−2028 compound, especially in lung H460 and prostate Du-145 cell lines with no change in the cytotoxic activity of this chemotherapeutic. Then, we demonstrated the mechanism of internalization of QD−*β*−CD(C−2028)−FA vehicles in all cell types studied. We achieved the drug release profile at an intracellular level and proved pH-dependent drug release from its nanoconjugates in the noncellular system.

The current work is the next step in the development of the QD−*β*−CD−FA platform for the efficient targeted delivery and release of UA drugs in cancer cells. Herein, we report an initial biological evaluation, the cell cycle progression, including induction of apoptosis, and cellular senescence as well as analysis of cell migration of our nanosystem for potential application in the treatment of lung and prostate cancers in humans. The biological response of QD−*β*−CD(C−2028)−FA and each of its components alone was assessed *in vitro* against human lung (H460) and prostate (Du-145 and LNCaP) cancer cells as well as against their respective (MRC-5 and PNT1A) normal cells.

## Materials and methods

### Materials

1-Ethyl-3-(3-dimethylaminopropyl)carbodiimide hydrochloride (EDC×HCl; product cat. no. E6383), 4-dimethylaminopyridine (DMAP; product cat. no. 8.51055), acetone (product cat. no. 179,124), diethyl ether (product cat. no. 673,811), dimethyl sulfoxide (DMSO; product cat. no. D8418), folic acid (FA; product cat. no. 1.03984), hydrochloric acid (HCl; product cat. no. 258,148), methanol (MeOH; product cat. no. 34,860), penicillin (product cat. no. P3032), phenol (product cat. no. 8.43984), streptomycin (product cat. no. S9137), trichloromethane (CHCl_3_; product cat. no. 366,927), X-gal (5-bromo-4-chloro-3-indolyl-β-D-galactosidase; product cat. no. B4252), and *β*−cyclodextrin (*β*−CD; product cat. no. C4805) were all purchased from Sigma-Aldrich (Merck KGa, Darmstadt, Germany). All used reagents and chemicals were of the highest purity available and used as received. Ultrapure water (conductivity 0.056 µS·cm^− 1^) was used to prepare all aqueous solutions (Milli-Q® IQ 7005 Water Purification System, Merck KGa).

C−2028 compound (9-{N-[(imidazo[4,5,1-de]-acridin-6-on-5-yl)aminopropyl]-N-methylaminopropylamino}-1’-nitroacridine), QD_green_, QD_green_−*β*−CD−FA, QD_green_−C−2028, and QD_green_−*β−*CD(C−2028)−FA vehicles were synthesized in-house. The materials required for their synthesis, as well as the structural evidence for all these compounds, were provided in the Supplementary information (Figures S1–S7).

### Methods

#### Preparation of C−2028: 9-{N-[(imidazo[4,5,1-de]-acridin-6-on-5-yl)aminopropyl]-N-methylaminopropylamino}-1’-nitroacridine×1.5 HCl

A mixture of derivative 5-{3-[N-(3-aminopropyl)-N-methylamino]propylamino}-imidazo[4,5,1-de]-acridin-6-one×2 HCl (0.001 mol), 5 mL phenol, and 9-phenoxy-1-nitroacridine (0.001 mol) was stirred at 90 °C for 24 h. After cooling, the reaction mixture was dissolved in methanol (MeOH) (~ 10 mL), poured into diethyl ether (~ 100 mL), and then stirred for 0.5 h. The precipitate was filtered off, washed with diethyl ether, and then with acetone. The product was dissolved in MeOH and a small quantity of silica gel was added and the solvent was evaporated. The remainder was loaded onto a dry chromatography column. The initial eluent was CHCl_3_ and then CHCl_3_/MeOH at a ratio of (15:1, 10:1 *v*/*v*), CHCl_3_/MeOH/NH_3_ (10:1:0.1 *v*/*v*); yield 45%, m.p. 207–209 °C. Procedures of the synthesis of derivatives: 5-{3-[N-(3-aminopropyl)-N-methylamino]propylamino}-imidazo[4,5,1-de]-acridin-6-one×2 HCl and 1-{3-[N-(3-aminopropyl)-N-methylamino]propylamino}-4-nitro-9(10 H)-acridone×2 HCl were described in the Supplementary information.

Elemental analysis: C_34_H_34_N_7_O_3_Cl_1,5_ × 2 H_2_O; electrospray ionization mass spectrometry (ESI-MS) C_34_H_31_N_7_O_3_ [M + 1]^+^ 586.258. ESI-MS analyses were performed on an Agilent 6500 Series Accurate-Mass Quadrupole-Time of Flight (Q-TOF) mass spectrometer (Agilent Technologies, Santa Clara, CA, USA). The system was controlled by Agilent MassHunter Workstation software (Agilent Technologies). ESI-MS spectrum for C−2028 was presented in Figure S1.

^1^H NMR (Me_2_SO-d_6_ + TFA) δ: 9.79 (s, 1H, N10*CH*); 9.70 (br.s, 1H, N1*H*CH_2_); 8.43 (d, J = 8.3 Hz, 1H, Ar-H); 8.37–8.41 (m, 1H, Ar-H); 8.36 (d, J = 7.8 Hz, 1H, Ar-H); 8.20 (d, J = 7.3 Hz, 1H, Ar-H); 8.08 (d, J = 8.8 Hz, 1H, Ar-H); 7.99–8.02 (m, 2 H, Ar-H); 7.92–7.99 (m, 2 H, Ar-H); 7.82 (d, J = 8.8 Hz, 1H, Ar-H); 7.63 (t, J = 7.6 Hz, 1H, Ar-H); 7.58 (t, J = 7.3 Hz, 1H, Ar-H); 7.01 (d, J = 9.3 Hz, 1H, C2); 3.58–3.63 (m, 2 H, C*H*_2_N9’H) 3.48–3.56 (m, 2 H, C*H*_2_N5H); 2.91–3.27 (m, 4 H, C*H*_*2*_NCH_3_C*H*_*2*_); 2.75 (s, 3 H, NCH_3_); 1.90–2.22 (m, 4 H, CH_2_C*H*_*2*_CH_2_N9’H; N5HCH_2_C*H*_*2*_CH_2_). ^1^H NMR spectra for C−2028 were presented in Figure S2.

#### Preparation of Ag_1.0_In_1.2_Zn_5.6_S_9.4_ nanocrystals (QD_green_)

The alloyed Ag−In−Zn−S quantum dots were prepared through the injection of sulfur dissolved in oleylamine (S/OLA) into a mixture of silver nitrate (AgNO_3_), indium (III) chloride (InCl_3_), zinc stearate, and 1-dodecanothiol (DDT) dissolved in 1-octadecene (ODE). This procedure allows for obtaining alloyed Ag−In−Zn−S quantum dots smoothly varying in composition via strict control of the precursor molar ratios. To stabilize the synthesized Ag−In−Zn−S quantum dots in a water solution, the initial hydrophobic ligands (stearic acid and 1-aminooctadecane) were exchanged for hydrophilic ligands: 11-mercaptoundecanoic acid (MUA). The quantum dots before and after (QD_green_) ligands exchange were spherical. Their diameter, as determined from transmission electron microscopy (TEM) images, were 3.2 ± 0.4 nm and 3.1 ± 0.6 nm respectively (Figure S3). In the obtained quantum dots (QD_green_), the inorganic core content (Ag_1.0_In_1.2_Zn_5.6_S_9.4_) was determined based on energy-dispersive X-ray spectroscopy (EDS) data (Figure S4). Hydrophilic quantum dots were labeled (QD_green_) due to their green luminescence (*λ*_max_ = 576 nm, Figure S5).

#### Preparation of QD_green_−β−CD−FA

The synthesis of the QD−*β*−CD−FA was performed according to the literature procedure [30]. In brief, in the first step, the QD−*β*−CD was synthesized using the formation of ester bonds between primary hydroxyl groups from *β*−CD and carboxyl functionalities on the surface of the QD. Then, using the formation of ester linkages between primary hydroxyl groups from *β*−CD and carboxyl functionalities of FA the QD−*β*−CD−FA nanoconjugate was formed.

#### Preparation of QD_green_−C−2028 and QD_green_− β−CD(C−2028)− FA

The two conjugates QD_green_−C−2028 and QD_green_− *β*−CD(C−2028)−FA were formed by non-covalent interactions. To attach the C−2028 molecules to the QD_green_ nanocrystals modified with 11-mercaptoundecanoic acid, the mixture of QD_green_ (2 mg·mL^−1^) and C−2028 (0.8 mM) in 0.02 M phosphate buffer pH 7.4 was first sonicated in a water bath for ca. 15 min and then stirred overnight in a ThermoMixer at room temperature. The as-obtained solution was dialyzed twice against 0.02 M PBS buffer pH 7.4 to remove unbound C−2028 molecules.

In turn, the formation of the QD_green_−*β*−CD(C−2028)−FA nanoconjugate took advantage of the properties of forming inclusion complexes in which the host is *β*−cyclodextrin while the guest is a potential anticancer drug (C−2028). The procedure for forming such a complex is very simple and involves preparing a mixture of QD_green_− *β*−CD−FA hybrid (1.0 mg·mL^− 1^) and C−2028 (300 µM), which was incubated overnight at room temperature. The unreacted mixture components were removed through a dialysis process against ultrapure water.

#### Cell culture

Three cancer cell lines and two normal cell lines were used to perform experiments in this work. The human non-small-cell lung cancer (H460), human prostate cancer (Du-145 and LNCaP), and human fetal lung fibroblasts (MRC-5) cell lines were purchased from the American Type Culture Collection (ATCC, USA). The human prostate PNT1A cell line was kindly provided by Prof. Jędrzej Antosiewicz from the Medical University of Gdańsk (Gdańsk, Poland). H460, LNCaP, and PNT1A cells were cultured in RPMI 1640 medium (Sigma-Aldrich, USA, product cat. no. R0883). Du-145 and MRC-5 cells were grown in an EMEM medium (Eagle’s Minimal Essential Medium, Sigma-Aldrich, USA, product cat. no. M2279). Both media were supplemented with 10% FBS (fetal bovine serum; Biowest, USA, product cat. no. S1520), 100 units·mL^− 1^ of penicillin (Sigma-Aldrich, Israel, product cat. no. P3032), and 100 µg·mL^− 1^ of streptomycin (Sigma-Aldrich, China, product cat. no. S9137). MRC-5 cells were cultured in an EMEM medium containing only 10% FBS without antibiotics. All cells grew were incubated in a humidified atmosphere and 5% CO_2_ at 37 °C according to the standard procedures.

#### Concentrations of the compounds in the experiments

The cytotoxic activity of all studied compounds against H460, Du-124, LNCaP, MRC-5, and PNT1A cells was examined previously by MTT assay [30]. In order to treat cells with the same concentration of the C−2028 compound, regardless of the form of its administration (unconjugated or conjugated form), corresponding to the estimated IC_80_ value for C−2028 alone following 72 h of incubation with cancer cells was chosen. The concentrations used in the experiments for C−2028, *β*−CD(C−2028), QD_green_−C−2028, and QD_green_−*β*− CD(C−2028)−FA nanoconjugates were 0.035 µM for H460 and MRC-5, 0.024 µM for Du-145, and 0.133 µM for LNCaP and PNT1A cell lines, respectively. The concentrations of different forms of nanoplatform (QD_green_, *β* −CD, and QD_green_−*β*−CD−FA), corresponding to the IC_80_ value of unbound C − 2028 compound in the nanoconjugates, were 1.2 µg·mL^− 1^ for H460 and MRC-5, 0.8 µg·mL^− 1^ for Du-145, and 4.4 µg·mL^− 1^ for LNCaP and PNT1A cell lines, respectively.

#### Cell cycle analysis

The PI/RNase Staining Kit (BD Pharmingen, Franklin Lakes, NJ, USA, product cat. no. 550,825) was used for DNA content analysis in accordance with the manufacturer’s recommendations. Briefly, the cells were seeded onto tissue culture plates and allowed to adhere overnight. After treatment the cells with QD_green_, QD_green_− *β*− CD− FA, C−2028, QD_green_−C−2028, and QD_green_−*β*−CD(C−2028)−FA for 24, 72, and 144 h at IC_80_ value, cells were harvested from the plates by incubation with a solution of trypsin, washed twice with PBS, fixed in ice-cold 80% ethanol, and stored overnight at − 20 °C. After centrifugation, the cells were stained with PI/RNase staining buffer (500 µL) at room temperature in the dark for 15 min and analyzed by flow cytometry (Accuri™ C6; Becton Dickinson, USA). The data were analyzed using BD Accuri™ C6 Software Version 1.0.264.21. Cell cycle analysis was performed at least three times.

#### Annexin V/propidium iodide (PI) binding assay

The FITC Annexin V Apoptosis Detection Kit I (BD Pharmingen, product cat. no. 556,547) was used to detect apoptosis, according to the manufacturer’s recommendations. In short, the cells were seeded onto tissue culture plates and allowed to adhere overnight. After treatment of the cells with compounds for the time indicated at IC_80_ value, cells were harvested from the plates by incubation with a solution of trypsin and washed twice with cold PBS. Next, the cells were resuspended in staining buffer (100 µL 1× binding buffer, 5 µL FITC Annexin V, and 5 µL propidium iodide (PI)) and incubated at room temperature in the dark for 15 min. The cells were then treated with 300 µL of 1× binding buffer and analyzed by flow cytometry as above. The analysis was performed at least three times and then included in the statistical analysis.

#### Senescence-associated β-galactosidase activity assay

Determination of cellular senescence required the use of pH 6.0-dependent β-galactosidase expression as a marker. H460, Du-145, and LNCaP cells were seeded at a plate with coverslips for 72 and 144 h of incubation, respectively, and incubated overnight. After treatment with QD_green_, *β*−CD, QD_green_−*β*−CD−FA, C− 2028, *β* −CD(C−2028), QD_green_−C−2028, and QD_green_−*β*−CD(C−2028)−FA for 72 and 144 h of incubation, cells were washed three times with PBS and then Fixative Solution (consisting of 0.2% glutaraldehyde and 2% formaldehyde in PBS) was added for 5 min. The cells were then washed three times with PBS and stained with a Staining Solution containing 1 mg·mL^− 1^ of X-gal. After incubation for 12–16 h at 37 °C, the cells were washed twice with PBS and observed by light microscope (Olympus BX60, Tokyo, Japan) using the Nomarski interference contrast at 200× magnification. The experiment was performed three times. For each repetition, 3–4 photos were taken and then included in the statistical analysis.

#### Wound healing migration assay

To analyze cell mobility, H460, and Du-145 cancer cells were seeded in a culture-insert (Ibidi culture-insert 2 well; Ibidi GmbH, Martinsried, Germany) at a density of 3 × 10^4^ cells per well. After allowing the cells to attach overnight, the culture-insert medium was removed and the cells were washed with RPMI or EMEM (for H460 or Du-145, respectively) to remove non-adherent cells. Then, the cells were incubated in a fresh medium containing QD_green_, QD_green_−*β*−CD−FA, C−2028, QD_green_−C−2028, and QD_green_− *β*−CD(C−2028)−FA at 0.25 IC_80_ value in an imaging chamber (cellVivo incubation system; Olympus, Tokyo, Japan) at 37 °C with 5% CO_2_. Images were captured every 6 h for 72 h under 100× magnification using a fluorescence microscope (IX83 Inverted Microscope; Olympus, Tokyo, Japan) connected to an XC50 digital color camera (Olympus, Tokyo, Japan). The experiment was performed three times. For each repetition, 2–3 photos were taken and then included in the statistical analysis. The percentage of wound closure was quantified with ImageJ software.

#### Statistical analysis

The data from all experiments were analyzed using the Kruskal−Wallis test for non-parametric data and Dunn’s test for post-hoc analysis. The results were presented as a median with a 95% confidence interval (CI), where a value of *p* ≤ 0.05 was considered significant. When the results were significant, Dunn’s test for post-hoc analysis was performed to check the influence of conjugation with the C−2028 compound. * *p* ≤ 0.05; ** *p* ≤ 0.01; *** *p* ≤ 0.01, **** *p* ≤ 0.0001 were considered as significant. The GraphPad Prism 8 (GraphPad Software Inc., USA) was used to perform statistical analysis.

## Results and discussion

QD_green_−*β*−CD(C−2028)−FA nanoconjugate used in this study was synthesized according to the procedure described in the Materials and methods section as well as in the Supplementary information. Fourier-transform infrared (FTIR) spectroscopy and TEM measurements were performed to confirm the introduction of C−2028 into the QD_green_−*β*−CD−FA conjugate and onto the QD_green_ surface. The representative FTIR spectra and TEM images are presented in Figure S6 and Figure S7 in the Supplementary information, respectively. The amount of loaded compound was determined based on UV-vis measurements of the C−2028 solutions before and after its interaction with QD_green_ and QD_green_−*β*−CD−FA nanoconjugates. It was found that the amount of C−2028 was ca. 19.0 mg and 6.16 mg per 1 g of QD_green_ and QD_green_−*β*−CD−FA, respectively. The physical parameters of the synthesized nanoconjugate are presented in Table 1.

**Table 1:** Size, polydispersity index (PDI), and zeta potential (ZP) of the QD_green_−*β*−CD−FA and QD_green_−*β*−CD(C−2028)−FA nanoconjugates. The parameters were estimated based on dynamic light scattering (DLS) measurements

	QD_green_−*β* −CD−FA	QD_green_− *β *−CD(C−2028)−FA
Size (hydrodynamic diameter)^1^	127 ± 15 nm	150 ± 20 nm
PDI	0.121	0.125
ZP	−30.0 mV	−33.4 mV

^1^size distribution based on intensity

### Cell cycle analysis

**Fig. 1:**
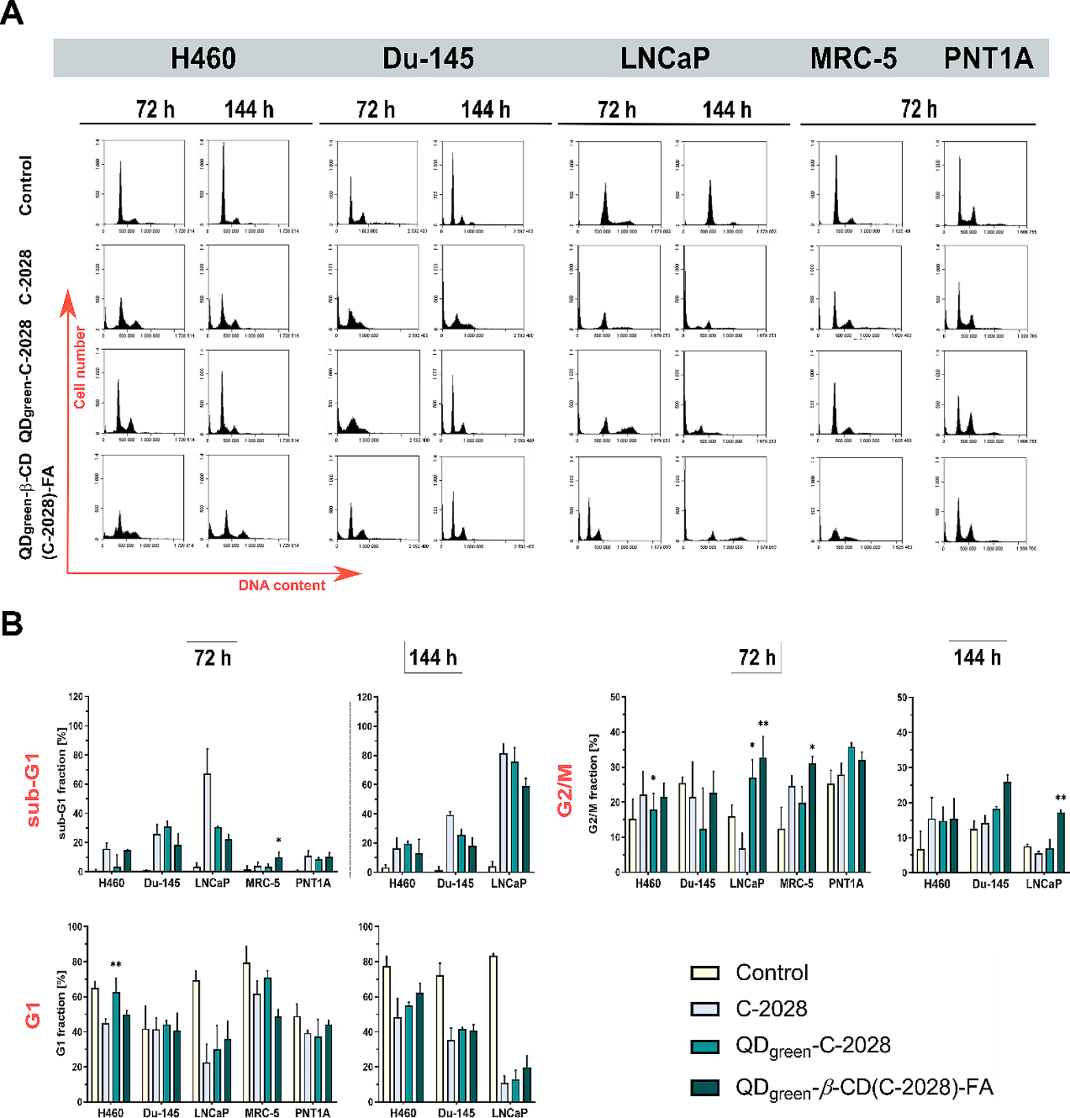
Cell cycle analysis of H460, Du-145, LNCaP, MRC-5, and PNT1A cells. (**A**) Representative plots of cells treated with C−2028, QD_green_−C−2028, and QD_green_−*β*−CD(C−2028)−FA. (**B**) Graphs represent the median of the percentage in the sub-G1, G1, and G2/M phases of the cell cycle in the studied cell lines with a 95% confidence interval (CI), where *p ≤ 0.05; **p ≤ 0.01 indicates statistically significant differences between the studied phases of cells incubated with C−2028 alone and its nanoconjugates. Statistical analysis was performed using the Kruskal–Wallis test for non-parametric data and Dunn’s test as a post-hoc test

C−2028 alone and its nanoconjugates were evaluated for their antiproliferative activity against three human cancer cell lines: H460, Du-145, and LNCaP, as well as two normal cell lines: MRC-5 and PNT1A. The details were presented in our previous paper [30] and in the Materials and methods section. Confocal images confirmed superior penetration as well as drug delivery in cancer cells treated with nanoconjugates [30, 31]. Therefore, the next step of our study was the biological evaluation of a novel folic acid-targeted receptor quantum dot−*β*− cyclodextrin carrier for unsymmetrical bisacridine (C−2028) in the treatment of human lung and prostate cancers. First, we evaluated cell cycle progression anomalies. It is known that cell apoptosis and cycle arrest are therapeutic targets for many chemotherapeutics [36, 37]. Many drugs have been shown to induce apoptosis and G2/M phase arrest [38, 39]. The impacts of C−2028 alone and its nanoconjugates (QD_green_−C−2028 and QD_green_−*β*−CD(C−2028)−FA) on the cell cycle progression of lung and prostate cancer and normal cells were measured by flow cytometry. As shown in Fig. 1 and Table S1 a–e, treatment of cancer cells with the C−2028 alone and its nanoconjugates resulted in a time-dependent increase in the population of hypodiploid cells (sub-G1 fraction), which represents apoptosis. This tendency was much higher in the case of cancer cells compared to normal cells, in which there was no obvious difference in control cells (untreated) and treated with compounds and their nanoconjugates (Table 2). Therefore, studied compounds are more toxic to cancer cells. The highest size of the sub-G1 population was observed in the case of prostate LNCaP cancer cells treated with the C−2028 compound (about 82%). Conjugation of this compound with nanoparticles decreased significantly in this population in the case of cancer cells, especially in LNCaP cells treated with QD_green_−*β*−CD(C−2028)−FA (82% vs. 57%). This dependence was higher in nanoconjugates with folic acid (QD_green_−*β*−CD(C−2028)−FA). In turn, the population of the G2/M phase in cells treated with the C−2028 compound remained at a similar level to control cells (untreated), except in LNCaP cells after 72 h of incubation, where the population size significantly decreased (16.2% for control vs. 6.8% for C−2028) and in MRC-5 cells, where the population size increased (17% vs. 23.9%, respectively). Conjugation of the C−2028 with nanoparticles may increase the size of the G2/M population. This tendency was observed mostly in the case of LNCaP and Du-145 cancer cell lines treated with conjugates with folic acid (QD_green_−*β*−CD(C−2028)−FA). In rest cell lines, especially in normal cell lines, there is no significant difference in the size of G2/M population cells treated with the C−2028 compound and its nanoconjugates. The G1 phase was the highest in all cell lines in the case of untreated cells (control). Treatment of cells with the C−2028 alone and its nanoconjugates resulted in a time-dependent decrease in the population of the G1 phase. This tendency was the highest in the case of cancer LNCaP cell lines. Conjugation of the C−2028 compound with QDs significantly increased the size of this population in the case of H460 cells (43.6% for C−2028 vs. 64.1% for QD_green_−C−2028) and LNCaP (22.6% for C−2028 vs. 37.6% for QD_green_−*β*−CD(C−2028)−FA). In turn, in normal MRC-5 cells treated with QD_green_−*β*−CD(C−2028)−FA the size of the G1 phase was lower than in cells treated with C−2028 alone (48.8% vs. 62.6%, respectively). Importantly, nanoparticles alone (QD_green_ and QD_green_−*β*−CD−FA) did not cause any changes in the cell cycle progression of the studied cancer H460, Du-145, and LNCaP cells, as well as normal MRC-5 and PNT1A cells (Fig. 1, Figure S8, and Table S1 a–e). Consequently, they are good carriers for transporting tested compounds.

**Table 2:** *P*-values from the Kruskal−Wallis test and the post-hoc Dunn’s multiple comparisons test for cell cycle analysis (sub-G1, G1, and G2/M fractions) in H460, Du-145, LNCaP, MRC-5, and PNT1A cells

Cell line	Phase	Incubation time [h]	Number of repetitions [*N*]	Kruskal–Wallis test	Dunn’s multiple comparisons test	
					C−2028* vs*. QD_green_− C−2028	C−2028 *vs.* QD_green_−*β*−CD (C−2028)−FA
H460	sub-G1	72	N1-N6 = 28	0.0003 ***	0.2692	> 0.9999
		144	N1-N6 = 24	0.0017 **	> 0.9999	> 0.9999
	G1	72	N1-N6 = 28	0.0065 **	0.0093 **	> 0.9999
		144	N1-N6 = 22	0.0055 **	> 0.9999	> 0.9999
	G2/M	72	N1-N6 = 28	0.0265 *	0.0417 *	0.9516
		144	N1-N6 = 21	0.0336 *	> 0.9999	> 0.9999
Du-145	sub-G1	72	N1-N6 = 20	0.0076 ***	> 0.9999	0.9210
		144	N1-N6 = 19	0.0056 **	> 0.9999	0.4115
	G1	72	N1-N6 = 20	0.0803	n/a	n/a
		144	N1-N6 = 19	0.0170 *	> 0.9999	> 0.9999
	G2/M	72	N1-N6 = 20	0.1842	n/a	n/a
		144	N1-N6 = 19	0.0156 **	0.4361	0.1286
LNCaP	sub-G1	72	N1-N6 = 16	0.0222 *	> 0.9999	0.4114
		144	N1-N6 = 17	0.0200 *	> 0.9999	0.5865
	G1	72	N1-N6 = 17	0.0232 *	> 0.9999	> 0.9999
		144	N1-N6 = 17	0.0141 *	> 0.9999	0.6640
	G2/M	72	N1-N6 = 17	0.0208 *	0.0166 *	0.0054 **
		144	N1-N6 = 17	0.0382 *	> 0.9999	0.0093 **
MRC-5	sub-G1	72	N1-N6 = 24	0.0303 *	0.5445	0.0310 *
	G1		N1-N6 = 24	0.0140 *	0.7235	0.8518
	G2/M		N1-N6 = 24	0.0031 **	> 0.9999	0.9751
PNT1-A	sub-G1	72	N1-N6 = 18	0.0171 *	> 0.9999	> 0.9999
	G1		N1-N6 = 18	0.1188	n/a	n/a
	G2/M		N1-N6 = 18	0.2628	n/a	n/a

Explanation of symbols: n/a, not applicable;* p*, significance level; *p ≤ 0.05; **p ≤ 0.01; ***p ≤ 0.001

### Induction of apoptosis

**Fig. 2:**
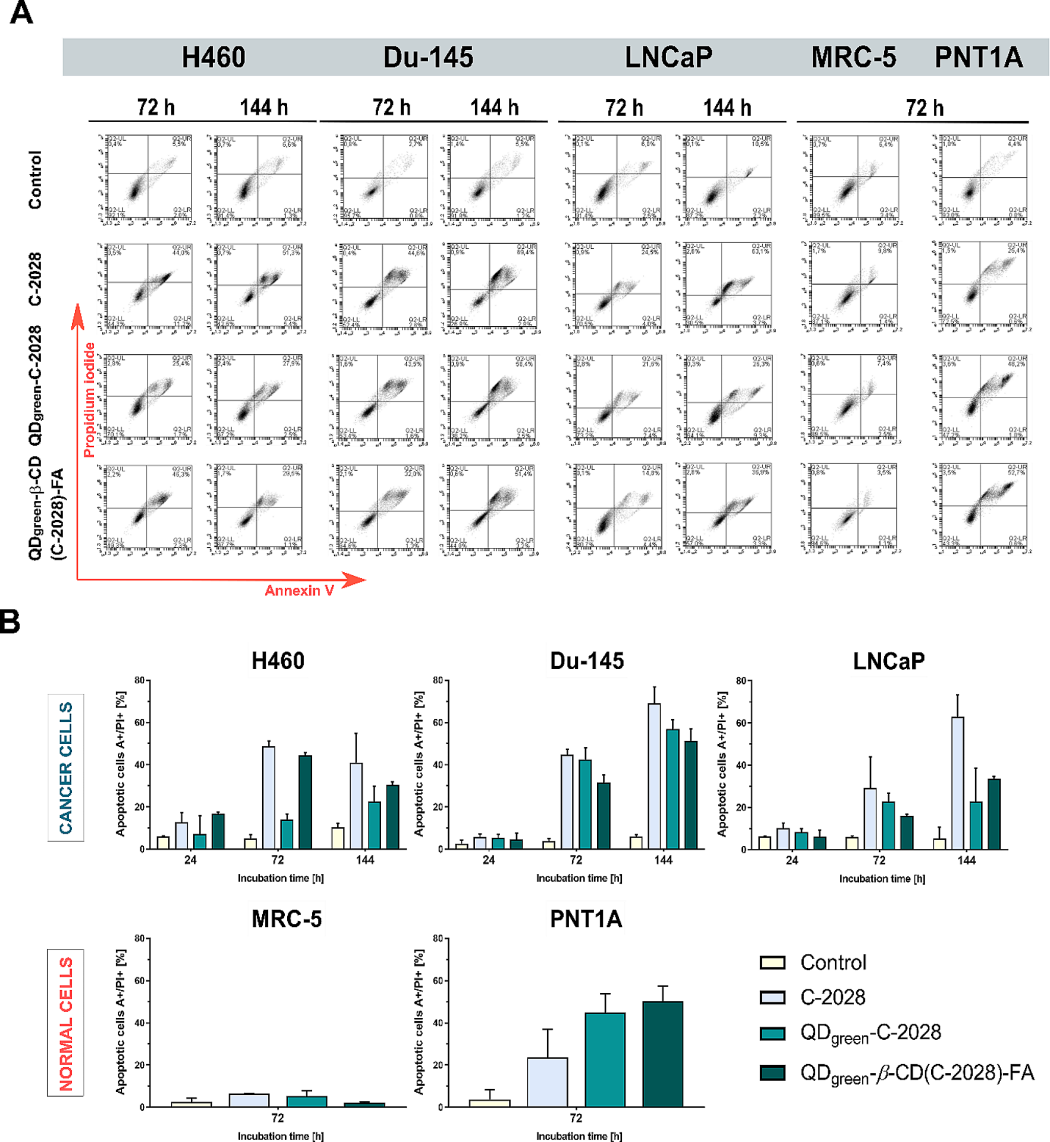
Flow cytometry analysis of phosphatidylserine externalization by Annexin V/propidium iodide (PI) assay in H460, Du-145, LNCaP, MRC-5, and PNT1A cells. (**A**) Representative plots of the studied cell lines treated with C−2028, QD_green_−C−2028, and QD_green_−*β*−CD(C−2028)−FA after treatment for 24, 72, and 144 h. (**B**) Graphs represent the median of the percentage of late apoptotic cells (A+/PI+) with a 95% confidence interval (CI). Statistical analysis between the fraction of late apoptotic cells incubated with C−2028 alone and its nanoconjugates was performed using the Kruskal–Wallis test for non-parametric data and Dunn’s test as a post-hoc test. The bottom left quadrant represents live cells (Annexin V negative, PI negative); the bottom right quadrant– early apoptotic cells (Annexin V positive, PI negative); the top right quadrant– late apoptotic cells (Annexin V positive, PI positive); top left quadrant– primary necrotic cells (Annexin V negative, PI positive)

The presence of the sub-G1 population in cancer cell lines treated with the C−2028 compound and its nanoconjugates (Fig. 1) prompted us to evaluate its apoptotic effects studied by flow cytometry. Figure 2A illustrates the dot plots of Annexin-V/propidium iodide staining of apoptotic cells. The C−2028 compound induced time-dependent apoptotic cell death in cancer cell lines, which is the strongest in the case of Du-145 cell lines (69.1%; Fig. 2, Figure S9, and Table S2 a–e). In the case of MRC-5 normal cells treated with C−2028, no obvious difference in the size of the late apoptosis cell population (A+/PI+) was observed (2.5% for control and 5.4% for C−2028). In contrast, in the second normal cell line - PNT1A, this was 4.0% vs. 23.8%, respectively. Conjugation of the C−2028 compound with nanoparticles decreased the late apoptosis cell population of all studied cancer cells (Table 3). This tendency was stronger in the case of QD_green_−C−2028 nanoconjugates (without folic acid in nanoconjugates). Interestingly, in the case of normal cell lines, the biological response was cell-line dependent. In normal human prostatic cell line PNT1A, the conjugation with nanoparticles increased the size of the population of late apoptosis cells (23.8% for C−2028, 44.9% for QD_green_−C−2028, and 50.3% for QD_green_−*β*−CD(C−2028)−FA). In this cell line, the use of FA in the conjugates increased the number of apoptotic cells in the population. In contrast, in the human fetal lung fibroblast cell line MRC-5, the size of the late apoptosis cells population was lower than 6% irrespective of the form of chemotherapeutic (alone or conjugated) − 5.4%, 4.9%, and 2.3% respectively. What is important, cancer, as well as normal cells treated with nanoparticles alone (QD_green_ and QD_green_−*β*−CD−FA), were viable and not undergoing apoptosis, which is consistent with cell cycle progression studies.

**Table 3:** *P*-values from the Kruskal−Wallis test and the post-hoc Dunn’s multiple comparisons test for phosphatidylserine externalization by Annexin V/propidium iodide (PI) assay in H460, Du-145, LNCaP, MRC-5, and PNT1A cells

Cell line		Incubation time [h]	Number of repetitions (*N*)	Kruskal–Wallis test	Dunn’s multiple comparisons test		
					C−2028 vs. QD_green_−C−2028	C−2028 vs. QD_green_−*β*−CD (C−2028)−FA	
H460		24	N1-N6 = 23	0.0115 *	0.5620	0.9755	
		72	N1-N6 = 26	0.0014 *	0.5081	> 0.9999	
		144	N1-N6 = 20	0.0047 **	0.3382	0.9897	
Du-145		24	N1-N6 = 18	0.1201	n/a	n/a	
		72	N1-N6 = 19	0.0126 *	> 0.9999	0.7608	
		144	N1-N6 = 17	0.0322 *	0.9794	0.7434	
LNCaP		24	N1-N6 = 18	0.6339	n/a	n/a	
		72	N1-N6 = 22	0.0040 **	0.9818	0.5156	
		144	N1-N6 = 21	0.0057 **	0.6461	0.8584	
MRC-5		72	N1-N6 = 20	0.2754	n/a	n/a	
PNTA-1		72	N1-N6 = 24	0.0119 *	0.8884	0.3867	

Explanation of symbols: n/a, not applicable; *p*, significance level; *p ≤ 0.05; **p ≤ 0.01

### Induction of cellular senescence

**Fig. 3:**
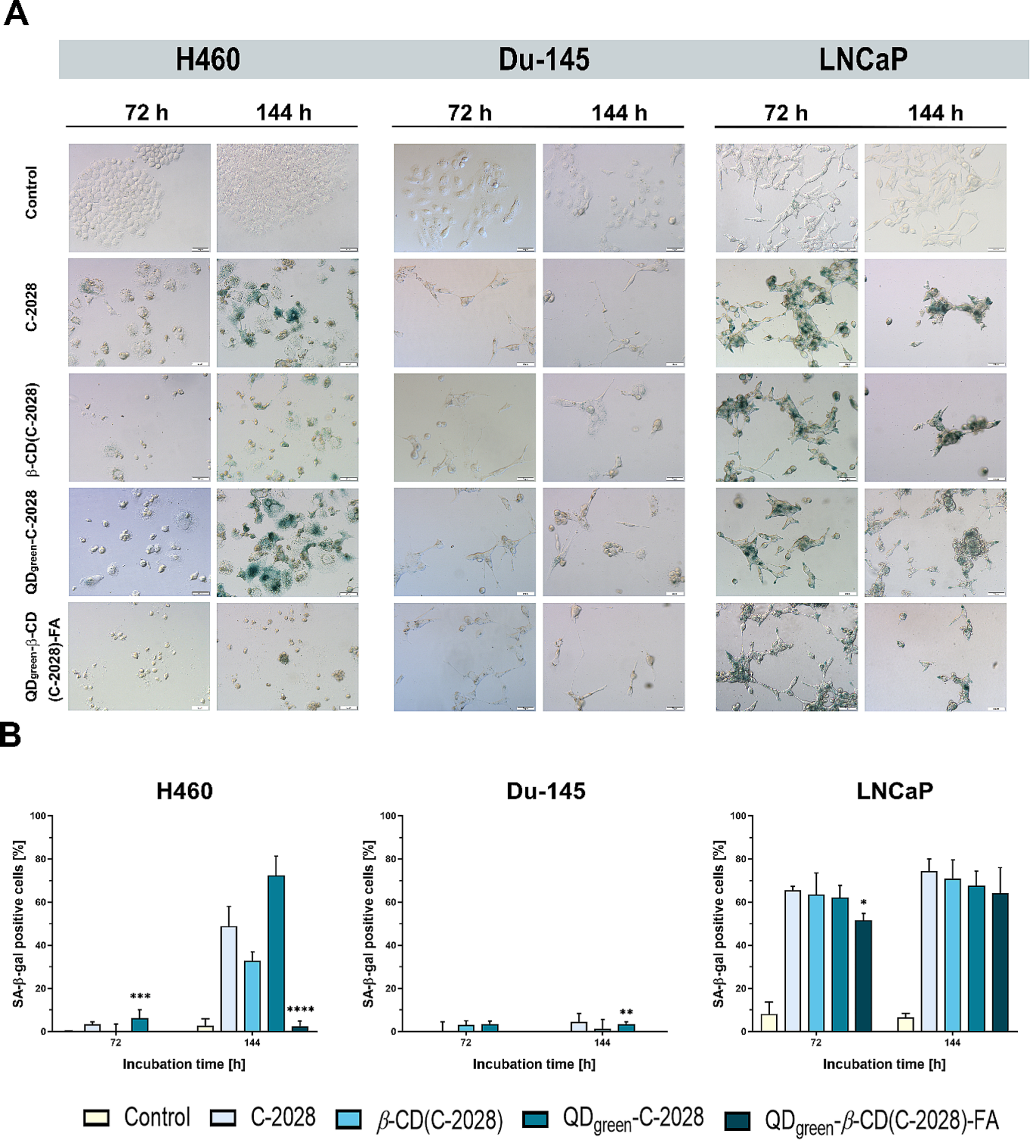
Cellular senescence of H460, Du-145, and LNCaP cancer cells following treatment with C−2028, *β*−CD(C−2028), QD_green_−C−2028, and QD_green_−*β*−CD(C−2028)−FA for the time indicated. Senescence-associated (SA) β-galactosidase (β-gal) activities were assessed by X-gal staining using a light microscope. (**A**) Representative images of the studied cells. (**B**) Graphs represent the median of the percentage of SA-β-gal positive cells with a 95% confidence interval (CI), where *p ≤ 0.05; **p ≤ 0.01; ***p ≤ 0.01, ****p ≤ 0.0001 indicate statistically significant differences between the studied phases of cells incubated with C−2028 alone and its nanoconjugates. Statistical analysis was performed using the Kruskal–Wallis test for non-parametric data and Dunn’s test as a post-hoc test. The scale bar is 50 μm

The C−2028 compound and its nanoconjugates induced apoptosis in studied cancer cell lines. However, this process concerns only part of the cancer cell population. Therefore, in the next part of our study senescence-associated-β-galactosidase (SA-β-gal) activity and morphological features (flattened cell shape and enlargement with abundant granulation) were evaluated as markers of senescence using bright-field microscopy (Fig. 3 and Figure S10). The typical blue color resulting from the metabolic activity of the SA-β-gal substrate was observed in two cell lines: H460 and LNCaP. In contrast, only a few percent of SA-β-gal positive cells in the population were observed in the Du-145 cancer cell line. In the case of H460 cells, the number of SA-β-gal positive cells was increased following prolonged incubation times (up to 144 h). The results show an increase for SA-β-gal positive cells after treatment with QD_green_−C−2028 (Table 4). Interestingly, the use of FA in this nanoconjugate (QD_green_−*β*−CD(C−2028)−FA) substantially decreased the senescence process to almost 3% (50.5% for C−2028, 74.2% for QD_green_−C−2028, and 2.7% for QD_green_−*β*−CD(C−2028)−FA). For LNCaP cells, the SA-β-gal positive cells remained at similar levels after 72 and 144 h of incubation. In this cell line, the number of SA-β-gal positive cells was similar for C−2028 and its nanoconjugates. A slight decrease in the number of senescent cells was detected only in the case of QD_green_−*β*−CD(C−2028)−FA nanoconjugates. In the prostate Du-145 cell line, only a few SA-β-gal positive cells were detected. The percentage of senescent cells never exceeded 4%. The conjugation of the C−2028 compounds has not changed biological response, however, even in this case the use of FA in nanoconjugates decreased the amount of SA-β-gal positive cells (4.0% for C−2028, 2.6% for QD_green_−C−2028, and 0.6% for QD_green_−*β*− CD(C−2028)−FA). In all of the studied cell lines treated with nanoconjugate containing folic acid as a linker (QD_green_−*β*−CD(C−2028)−FA), the reduction of the senescence process was observed, especially in the case of H460 cells (50.1% vs. 2.7% for C−2028 alone and QD_green_−*β*−CD(C−2028)−FA, respectively). Zhang X. et al. showed FA as a vitamin to oppose telomere dysfunction, as well as the associated senescence phenotype induced by sleep deprivation [40]. Therefore, that can be the reason for the reduced number of senescent cells after treatment with QD_green_−*β*−CD(C−2028)−FA nanoconjugates. Furthermore, nanoparticles alone (QD_green_, *β*−CD, and QD_green_−*β*−CD−FA) did not induce senescence in all of the studied cancer cells after 144 h of incubation, which is consistent with the above results (Figure S10).

**Table 4:** *P*-values from the Kruskal−Wallis test and the post-hoc Dunn’s multiple comparisons test for senescence assay (expressed as % of SA-β-gal positive cells in the population) in H460, Du-145, and LNCaP cells

Cell line	Incubation time [h]	Number of repetitions (*N*)	Kruskal–Wallis test	Dunn’s multiple comparisons test	
				C−2028 vs. QD_green_−C−2028	C−2028 vs. QD_green_−*β*−CD (C−2028)−FA
H460	72	N1-N6 = 60	< 0.0001 ****	0.2545	0.0005 ***
	144	N1-N6 = 60	< 0.0001 ****	0.0678	< 0.0001 ****
Du-145	72	N1-N6 = 63	0.0007 ***	0.1467	0.5040
	144	N1-N6 = 62	0.0028 **	0.7297	0.0045 **
LNCaP	72	N1-N6 = 53	< 0.0001 ****	> 0.9999	0.0235 *
	144	N1-N6 = 62	< 0.0001 ****	0.2527	0.1629

Explanation of symbols: n/a, not applicable; *p*, significance level; *p ≤ 0.05; **p ≤ 0.01; ***p ≤ 0.001; ****p ≤ 0.0001

### Wound healing migration assay

**Fig. 4:**
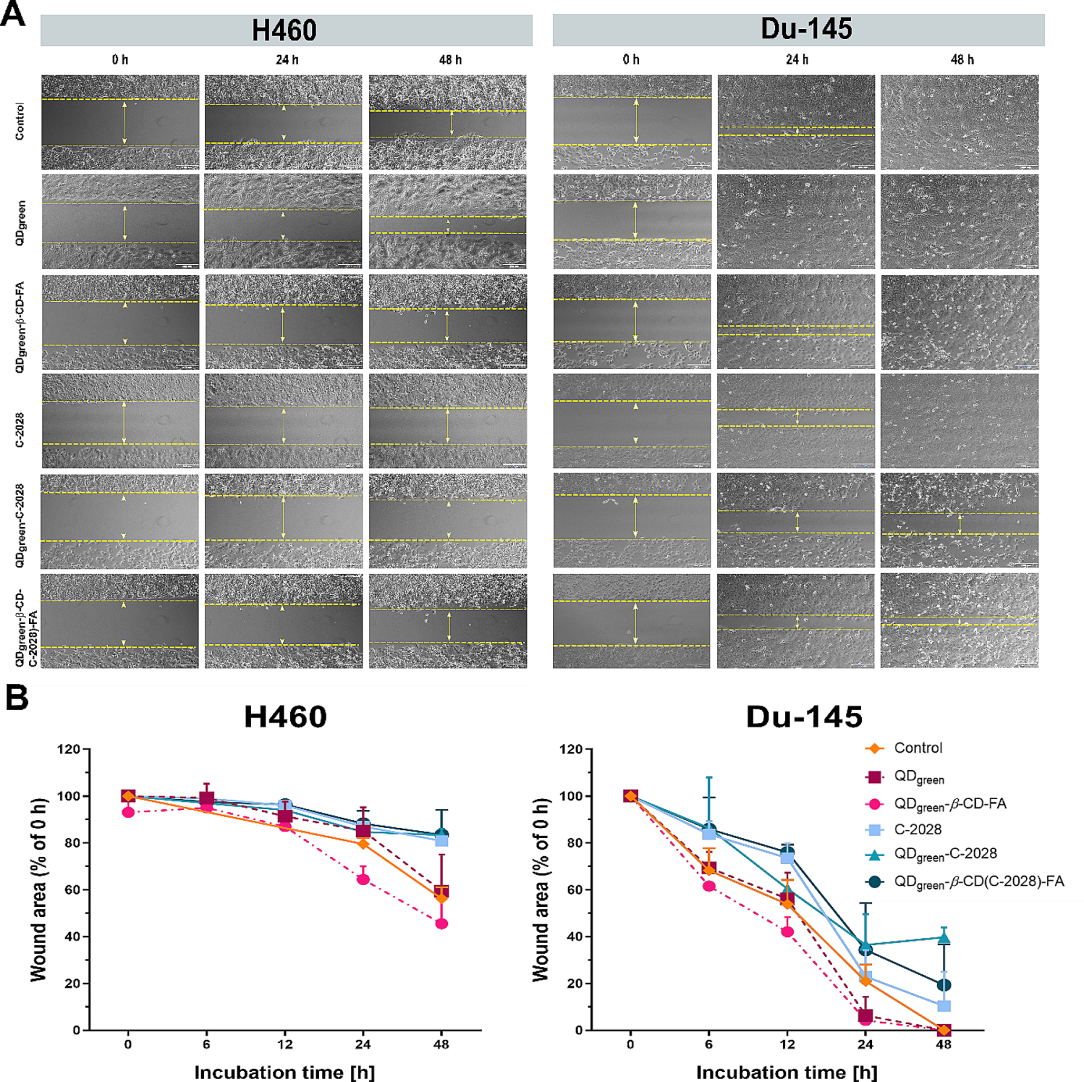
Analysis of cell migration by *in vitro* wound healing migration assay of H460 and Du-145 cancer cells following treatment with QD_green_, QD_green_−*β*−CD−FA, C−2028, QD_green_−C−2028, and QD_green_−*β*−CD(C−2028)−FA. (**A**) Time-lapse microscopy representative images after culture insert removal. (**B**) Graphs represent the differences between wound area (% of 0 h) cells incubated with C−2028 alone and its nanoconjugates with a 95% confidence interval (CI). Statistical analysis between the fraction of late apoptotic cells incubated with C−2028 alone and its nanoconjugates was performed. The scale bar is 200 μm

The next step of our study was to evaluate the anti-migratory potential of the studied nanoconjugates, thus, the wound healing migration assay was performed on H460 and Du-145 cancer cell lines. As shown in Fig. 4, cells treated with nanoplatforms (QD_green_ and QD_green_−*β−*CD−FA) gradually repopulated the wound area in a time-dependent manner in both cancer cell lines. This area was getting smaller slightly faster in the case of cells treated with nanoparticles, compared to the control group. In contrast, the C−2028 compound significantly reduced the migration of cells (Table 5). Conjugation of C−2028 with nanoparticles in prostate Du-145 cancer cells further enhanced this effect, which was stronger in the case of QD_green_−C−2028. This effect was retained during the entire exposure time, with the highest difference during 24–48 h compared to that in the control. In the case of the lung H460 cancer cell line, the weak migratory effect was observed. These cells tended to grow in layers, which was observed mostly in the case of the control group and cells treated with nanoplatforms. However, in the case of the C−2028 compound and its nanoconjugates decrease in the rate of cell migration was observed. It is important to note that the concentrations of all studied compounds were slightly cytotoxic which were 0.25 of IC_80_ values. This may suggest that the observed anti-migratory properties of C−2028 and its nanoconjugates were not affected by their cytotoxic effect.

### Conclusions and future considerations

To conclude, anticancer drug delivery carriers with a controllable drug release have desirable advantages in efficiency, selectivity, safety, tolerance, and side effects. In this study, we presented that the anticancer C−2028 compound alone and its nanoconjugates induced time-dependent apoptosis, mostly in cancer cells. However, the linkage of this compound with nanoplatforms (QD_green_ and QD_green_−*β*−CD−FA) decreased the number of late apoptotic cells in cancer cell lines. In the case of normal cell lines, the biological response was cell-line dependent. The apoptotic cells, after treatment with C−2028 and its nanoconjugates were observed in the PNT1A cell line, in contrast to the MRC-5 cell line. Similar results for normal cell lines were observed in cell cycle studies. The C−2028 compound and its nanoconjugates did not induce apoptosis (sub-G1 population) in the normal MRC-5 cell line, in contrast to the PNT1-A cell line. Unsymmetrical bisacridine and its nanoconjugates induced cellular senescence, depending on the cell line. This process was observed in cancer H460 and LNCaP cell lines in opposition to the Du-145 cancer cell line. Moreover, the senescence process can be also dependent on the composition of nanoconjugates, especially in the case of H460 cancer cells. The strongest effect was observed in cells treated with QD_green_−C−2028 nanoconjugates, while only a few percent of SA-β-gal positive cells after treatment with QD_green_−*β*−CD(C−2028)−FA nanoconjugates were detected. The wound healing migration assay showed that the C−2028 compound significantly reduced the migration of cells. Conjugation of C−2028 with nanoparticles further enhanced this effect, especially in the case of QD_green_−C−2028 in prostate Du-145 cancer cells. Nanoplatforms alone (QD_green_ and QD_green_−*β*−CD−FA) did not affect the processes of apoptosis and senescence in all of the studied cancer and normal cells. In summary, the described results demonstrate the high potential of a novel folic acid-targeted receptor quantum dot−*β*−cyclodextrin carrier (QD_green_−*β*−CD−FA) for drug delivery in cancer treatment.

**Table 5:** *P*-values from the Kruskal−Wallis test and the post-hoc Dunn’s multiple comparisons test for cell migration by *in vitro* wound healing migration assay of H460 and Du-145 cancer cells (significant differences between wound area (% of 0 h) cells incubated with C−2028 alone and its nanoconjugates) in H460, Du-145, and LNCaP cells

Cell line	Incubation time [h]	Number of repetitions (*N*)	Kruskal–Wallis test	Dunn’s multiple comparisons test			
				C−2028 vs. QD_green_−C−2028	C−2028 vs. QD_green_−*β*−CD(C−2028)−FA		
H460	0	N1-N6 = 25	n/a	n/a	n/a		
	6	N1-N6 = 6	> 0.9999	n/a	n/a		
	12	N1-N6 = 6	0.6667	n/a	n/a		
	24	N1-N6 = 25	0.0196 *	> 0.9999	> 0.9999		
	48	N1-N6 = 60	0.0028 **	> 0.9999	> 0.9999		
Du-145	0	N1-N6 = 24	n/a	n/a	n/a		
	6	N1-N6 = 24	0.0172 *	> 0.9999	> 0.9999		
	12	N1-N6 = 21	0.0209 *	0.7349	> 0.9999		
	24	N1-N6 = 35	0.0003 ***	0.6296	> 0.9999		
	48	N1-N6 = 33	< 0.0001 ****	0.1674	0.9299		

Explanation of symbols: n/a, not applicable; *p*, significance level; *p ≤ 0.05; **p ≤ 0.01; ***p ≤ 0.001; ****p ≤ 0.0001

The results obtained so far will be used to develop further nanoconjugates. In the future, we would like to test different targeting linkers (e.g., transferrin) that would allow for the selective delivery of drugs to cancer cells. We also plan to perform *in vivo* experiments for QD_green_−*β*−CD(C−2028)−FA nanoconjugates and new platforms to verify the performance of the proposed drug delivery system in a physiological environment.

### Electronic supplementary material

Below is the link to the electronic supplementary material.

Supplementary Material 1

## Data Availability

The datasets generated during and/or analyzed during the current study are available from the corresponding author upon reasonable request.

## Abbreviations

C−2028: 9-{N-[(imidazo[4,5,1-de]-acridin-6-on-5-yl)aminopropyl]-N-methylaminopropylamino}-1’-nitroacridine
FA: Folic acid
FRs: Folic acid receptors
FTIR: Fourier-transform infrared spectroscopy
NPs: Nanoparticles
QDs: Quantum dots, Ag−In−Zn−S nanocrystals
QD−*β*−CD−FA: Quantum dots−*β*−cyclodextrin−folic acid (nanoplatforms)
SA-β-gal: Senescence-associated-β-galactosidase
TEM: Transmission electron microscopy
TME: Tumor microenvironment
UA: Unsymmetrical bisacridine
X-gal: 5-Bromo-4-chloro-3-indolyl-β-D-galactosidase
β−CD: *β*−Cyclodextrin
