# Supplementary material for: In vitro biological evaluation of a novel folic acid-targeted receptor quantum dot−β−cyclodextrin carrier for C−2028 unsymmetrical bisacridine in the treatment of human lung and prostate cancers
Source: Pharmacol Rep. 2024 Jun 18;76(4):823–37. doi: 10.1007/s43440-024-00606-4 (PMC11294431; doi:10.1007/s43440-024-00606-4)
Supplement: Supplementary file 1 — Supplementary Material 1 [file 43440_2024_606_MOESM1_ESM.docx]

***Supplementary information***

***for***

***In vitro* biological evaluation of a novel folic acid-targeted receptor quantum dot−*β*−cyclodextrin carrier for C−2028 unsymmetrical bisacridine in the treatment of human lung and prostate cancers**

**Joanna Pilch ^1*^, Agnieszka Potęga ^1^, Patrycja Kowalik ^2^, Agata Kowalczyk ^3^,**

**Piotr Bujak ^4^, Artur Kasprzak ^4^, Ewa Paluszkiewicz ^1^, and Anna Maria Nowicka ^3^**

^1^ Faculty of Chemistry, Gdańsk University of Technology, Gdańsk, Poland

^2^ Institute of Physical Chemistry, Polish Academy of Science, Warsaw, Poland

^3^ Faculty of Chemistry, University of Warsaw, Warsaw, Poland

^4^ Faculty of Chemistry, Warsaw University of Technology, Warsaw, Poland

***** Corresponding author: Faculty of Chemistry, Gdańsk University of Technology, Narutowicza 11/12 Str., 80-233 Gdańsk, Poland, e-mail: [joanna.pilch@pg.edu.pl](mailto:joanna.pilch@pg.edu.pl); phone: +48 58 347 12 97

**Materials**

Acetone (product cat. no. 179124), dimethyl sulfoxide (DMSO; product cat. no. D8418),
1-dodecanethiol (DDT, 98%; product cat. no. 471364), formic acid (product cat. no. 695076), hydrochloric acid (HCl; product cat. no. 258148), indium(III) chloride (InCl_3_, 98%; product cat. no. 334065), methanol (MeOH; product cat. no. 34860), 11-mercaptoundecanoic acid (MUA; product cat. no. 674427), oleylamine (OLA, 70%; product cat. no. O7805), silver nitrate (AgNO_3_, 99%; product cat. no. 209139), sodium hydroxide (98%; product cat. no. S5881), sulfur (99%; product cat. no. 13825), 1-octadecene (ODE, 90%; product cat. no. O806), zinc stearate (technical grade; product cat. no. 307564) were supplied by Sigma-Aldrich (Merck KGa, Darmstadt, Germany).

**Methods**

*Preparation of derivative 5-{3-[N-(3-aminopropyl)-N-methylamino]propylamino}-imidazo[4,5,1-de]-acridin-6-one×2 HCl*

The previously obtained derivative 1-{3-[N-(3-aminopropyl)-N-methylamino]propylamino}-4-nitro-9(10H)-acridone×2 HCl (0.0027 mol), 10% Pd/C (catalytic quantities), and 40 mL 96% formic acid were hydrogenated by passing gaseous hydrogen through them at room temperature for 24 h. After this time, the catalyst was filtered off, and to the filtrate was added 2-3 mL concentrated HCl, and the mixture was heated at 110 °C for 24 h. The formic acid was evaporated, and the resulting remainder was heated for 3 h in a water-methanol mixture at a ratio of 1/1 (about 50 mL). The solvent was evaporated, and the remainder was dissolved in methanol and acidified with concentrated HCl. The product was crystallized from acetone; yield 74%.

*Preparation of derivative 1-{3-[N-(3-aminopropyl)-N-methylamino]propylamino}-4-nitro-9(10H)-acridone×2 HCl*

A mixture of 1-chloro-4-nitro-9(10H)-acridone (0.01 mol), 3,3-diamino-N-methyldipropylamine (0.04 mol) in DMSO (50 mL) was stirred at room temperature for 3 h. After this time, water was added (~200 mL) and then stirred for 10 min. The precipitate was collected by filtration and suspended into water (~100 mL), and then acidified with a dilute HCl and stirred again for 15 min. The insoluble precipitate was filtered off, and the filtrate was evaporated to a smaller volume. The product was precipitated out using acetone (~100 mL), and then was filtered off; yield 81%.

*Synthesis of Ag*−*In*−*Zn*−*S quantum dots (QD_green_)*

All operations were carried out under a constant dry argon flow. AgNO_3_ (0.03 g, 0.17 mmol), InCl_3_ (0.13 g, 0.59 mmol), zinc stearate (0.87 g, 1.37 mmol), and DDT (0.20 g, 1.00 mmol) were mixed with ODE (15 mL) in a three-neck flask. The mixture was heated to 150 °C until a homogenous solution was formed. Then sulfur (0.015 g, 0.47 mmol) dissolved in 1 mL of OLA was quickly injected into the reaction solution. The temperature was increased to 180 °C, and the mixture was kept at this temperature for 60 min. After the mixture was cooled to room temperature, toluene (20 mL) was added, and the reaction mixture was centrifuged - the isolated black precipitate was separated. The supernatant was treated with 30 mL of acetone leading to the precipitation of the desired fraction of Ag-In-Zn-S quantum dots. The Ag−In−Zn−S quantum dots were separated by centrifugation (7000 rpm, 5 min) and then redispersed in toluene.

*Preparation (ligand exchange) of hydrophilic Ag*−*In*−*Zn*−*S quantum dots (QD_green_)*

All operations were carried out under a constant dry argon flow. A mixture of MUA (0.5 g, 2.3 mmol) and NaOH (0.1 g, 2.5 mmol) in water (10 mL) was heated with stirring at 50 °C until a homogenous solution was formed. Then, a toluene dispersion (10 mL) of Ag−In−Zn−S quantum dots capped with initial ligands was injected into this solution. The as-obtained two-phase mixture was heated at 80 °C for 8 h under argon. After cooling, the reaction mixture was centrifuged to obtain a complete phase separation; the solid and the organic phases were discarded. The aqueous phase was then mixed with 20 mL of acetone, resulting in the precipitation of QD_green_. After centrifugation, the QD_green_ was redispersed in 10 mL of water.

*Characterization*

Elemental analysis was performed with a multichannel Quantax 400 energy-dispersive X-ray spectroscopy (EDS) system with a 125 eV xFlash detector 5010 (Bruker) using a 15 kV electron beam energy. Transmission electron microscopy (TEM) studies were performed on a Zeiss Libra 120 electron microscope operating at 120 kV. UV-vis-NIR spectra were registered using a Cary 5000 (Varian) spectrometer. Steady-state PL spectra of water dispersion of samples were measured with an FLS-980 fluorescence spectrophotometer equipped with a 450 W Xe lamp and photomultiplier (Hamamatsu, R928P) detector with a standard 10 mm cuvette (λ_exc_ = 375 nm).

**
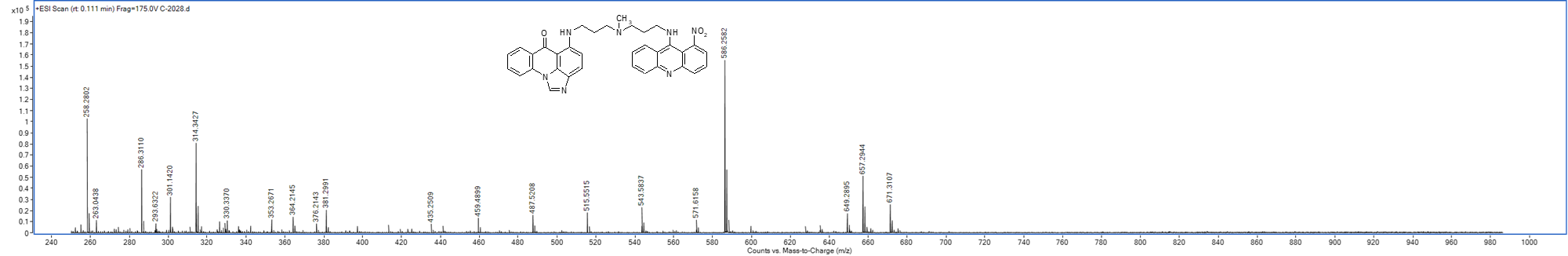
**

**Figure S1.** ESI-MS spectrum for C−2028. *m*/*z* [M + H]^+^ calculated for [C_34_H_31_N_7_O_3_]: 585.6550; found: 586.2582. The chemical structure of compound C−2028 is also included.

**
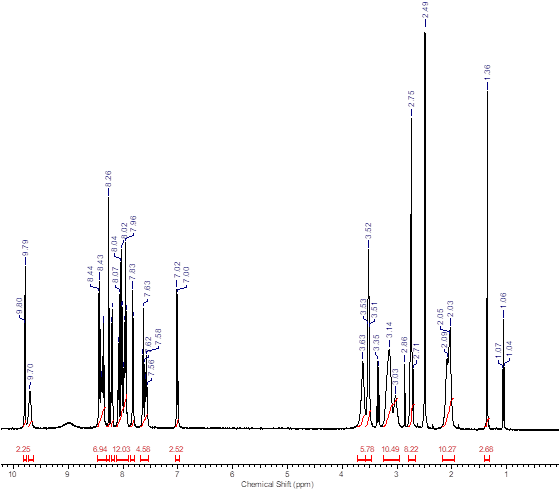
**

**
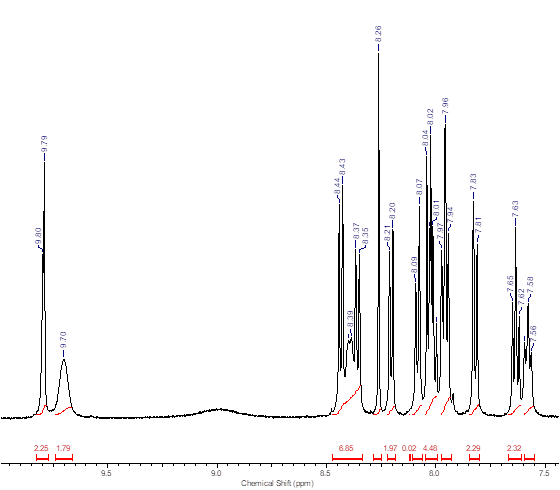
**

**Figure S2.** ^1^H NMR spectra for C−2028.


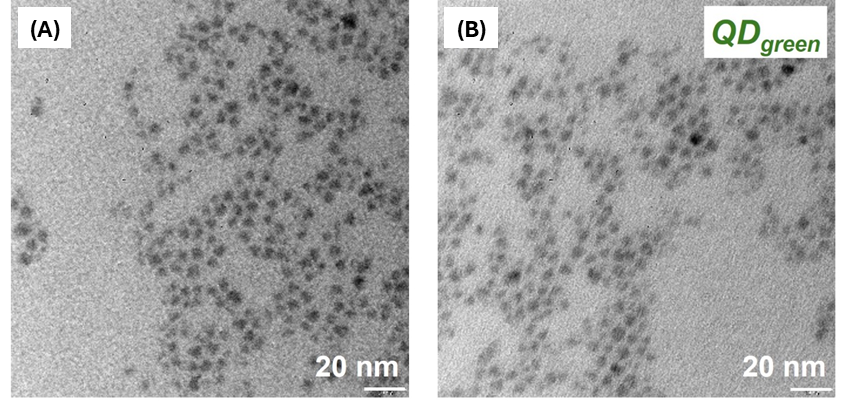


**Figure S3.** Transmission electron microscopy (TEM) images of Ag−In−Zn−S quantum dots before (**A**) and after (**B**) the exchange of initial capping ligands for 11-mercaptoundecanoic acid (MUA).


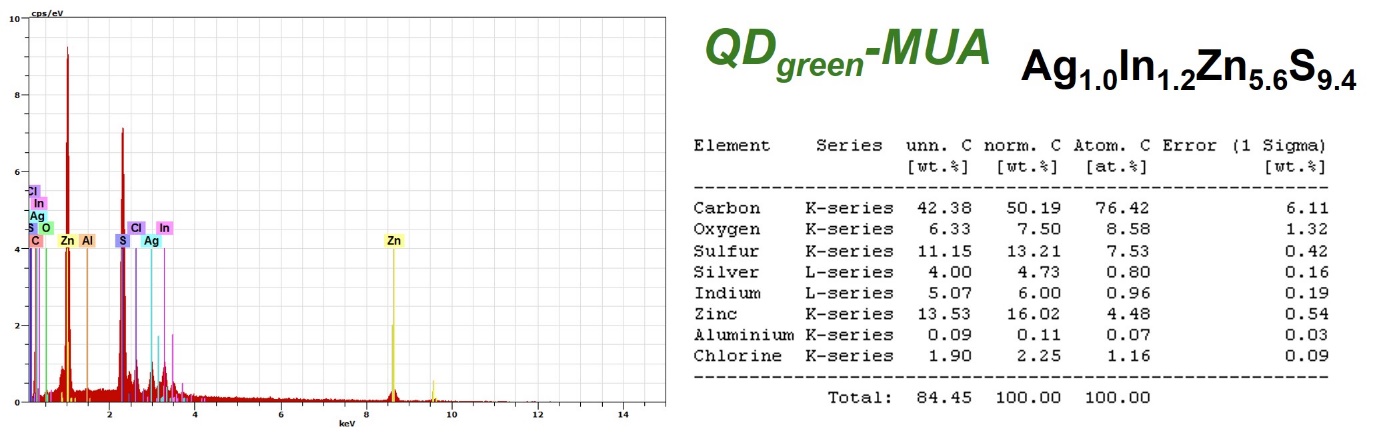


**Figure S4.** Energy-dispersive spectrum of Ag_1.0_In_1.2_Zn_5.6_S_9.4_ quantum dots (QD_green_).


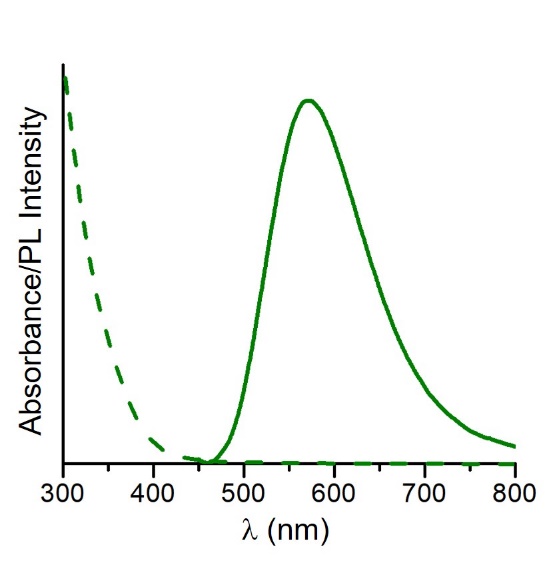


**Figure S5.** Absorbance (dash line) and emission (solid line) of water dispersion of Ag_1.0_In_1.2_Zn_5.6_S_9.4_ quantum dots (QD_green_).

**Figure S6.** Transmission electron microscopy (TEM) images of QD_green_, QD_green_−C−2028, QD_green_−*β*−CD−FA, and QD_green_−*β*−CD(C−2028)−FA.

**Figure S7.** Representative Fourier-transform infrared spectroscopy (FTIR) spectra of QD_green_−*β*−CD(C−2028)−FA nanoconjugate and its pure components.

**Table S1 a.** Analysis of cell cycle by flow cytometry in H460 cells. Data shows the percentage of cells treated with QD_green_, QD_green_−*β*−CD−FA, C−2028, QD_green_−C−2028, and QD_green_−*β*−CD(C−2028)−FA nanoconjugates at IC_80_ value for the time indicated in the sub-G1, G1, S, G2/M, and Poli (polyploid cells) phases of the cell cycle. Data represented the averages of three independent experiments.

| **Compound** |  | **Phases of the cell cycle** | | | | | | | | | |
| --- | --- | --- | --- | --- | --- | --- | --- | --- | --- | --- | --- |
|  | **[h]** | **sub-G1** | **±** | **G1** | **±** | **S** | **±** | **G2/M** | **±** | **Poli** | **±** |
| **Control** | 24 | 2.2 | 1.8 | 59.4 | 2.1 | 15.5 | 2.0 | 21.0 | 0.9 | 2.0 | 0.8 |
|  | 72 | 1.0 | 0.4 | 64.8 | 2.7 | 14.4 | 1.5 | 16.2 | 2.9 | 3.6 | 2.8 |
|  | 144 | 3.7 | 1.0 | 78.0 | 3.3 | 4.8 | 0.8 | 7.8 | 3.2 | 5.7 | 1.3 |
| **QD_green_** | 24 | 0.8 | 0.1 | 59.5 | 1.6 | 14.9 | 3.6 | 20.8 | 2.9 | 4.0 | 2.7 |
|  | 72 | 1.0 | 0.3 | 6.4 | 4.5 | 11.0 | 1.2 | 15.8 | 0.7 | 5.8 | 5.1 |
|  | 144 | 3.0 | 0.9 | 74.6 | 6.3 | 6.4 | 2.9 | 11.0 | 3.5 | 4.9 | 2.3 |
| **QD_green_−*β*−CD−FA** | 24 | 1.5 | 0.6 | 60.6 | 1.0 | 12.0 | 0.1 | 22.0 | 0.2 | 4.0 | 1.4 |
|  | 72 | 0.9 | 0.1 | 63.6 | 1.3 | 10.4 | 2.2 | 18.2 | 0.4 | 6.9 | 3.7 |
|  | 144 | 1.8 | 0.3 | 75.1 | 4.6 | 6.5 | 2.1 | 12.8 | 1.6 | 3.8 | 3.6 |
| **C−2028** | 24 | 3.9 | 1.3 | 36.9 | 6.9 | 28.0 | 4.7 | 29.0 | 4.3 | 2.2 | 2.2 |
|  | 72 | 16.3 | 2.9 | 43.6 | 4.8 | 11.6 | 0.8 | 23.3 | 4.0 | 5.3 | 2.1 |
|  | 144 | 18.9 | 4.1 | 49.0 | 9.9 | 10.6 | 2.3 | 16.2 | 4.9 | 5.4 | 2.5 |
| **QD_green_−C−2028** | 24 | 2.7 | 0.7 | 38.7 | 6.4 | 15.5 | 4.9 | 40.2 | 5.8 | 2.8 | 3.9 |
|  | 72 | 5.7 | 3.3 | 64.1 | 5.0 | 9.4 | 1.4 | 17.2 | 3.5 | 3.5 | 2.1 |
|  | 144 | 18.7 | 3.0 | 54.5 | 2.8 | 6.7 | 1.7 | 16.1 | 2.3 | 4.0 | 0.8 |
| **QD_green_−*β*−CD(C−2028)−FA** | 24 | 7.3 | 4.0 | 38.6 | 6.9 | 14.8 | 3.2 | 37.8 | 10.4 | 1.5 | 0.5 |
|  | 72 | 15.0 | 0.1 | 49.6 | 3.6 | 9.9 | 0.1 | 21.5 | 5.6 | 4.2 | 2.0 |
|  | 144 | 15.7 | 6.0 | 58.6 | 11.1 | 5.8 | 0.8 | 16.2 | 4.6 | 3.7 | 0.5 |

**Table S1 b.** Analysis of cell cycle by flow cytometry in Du-145 cells. Data shows the percentage of cells treated with QD_green_, QD_green_−*β*−CD−FA, C−2028, QD_green_−C−2028, and QD_green_−*β*−CD(C−2028)−FA nanoconjugates at IC_80_ value for the time indicated in the sub-G1, G1, S, G2/M, and Poli (polyploid cells) phases of the cell cycle. Data represented the averages of three independent experiments.

| **Compound** |  | **Phases of the cell cycle** | | | | | | | | | |
| --- | --- | --- | --- | --- | --- | --- | --- | --- | --- | --- | --- |
|  | **[h]** | **sub-G1** | **±** | **G1** | **±** | **S** | **±** | **G2/M** | **±** | **Poli** | **±** |
| **Control** | 24 | 0.5 | 0.3 | 52.3 | 5.5 | 8.6 | 0.7 | 28.6 | 2.9 | 10.0 | 3.5 |
|  | 72 | 0.9 | 0.6 | 45.9 | 7.8 | 6.5 | 0.9 | 25.5 | 1.6 | 21.1 | 8.9 |
|  | 144 | 2.0 | 1.6 | 71.3 | 8.3 | 3.7 | 1.0 | 12.4 | 2.4 | 10.7 | 7.0 |
| **QD_green_** | 24 | 0.5 | 0.5 | 47.5 | 4.2 | 9.3 | 1.1 | 30.2 | 3.0 | 12.5 | 2.9 |
|  | 72 | 1.3 | 0.8 | 55.9 | 5.3 | 5.9 | 1.3 | 24.4 | 3.1 | 12.5 | 3.2 |
|  | 144 | 2.3 | 2.2 | 71.9 | 4.4 | 2.8 | 2.3 | 13.6 | 1.5 | 9.4 | 6.2 |
| **QD_green_−*β*−CD−FA** | 24 | 0.5 | 0.4 | 51.9 | 3.1 | 7.9 | 2.0 | 30.0 | 2.9 | 9.7 | 0.8 |
|  | 72 | 1.7 | 0.2 | 52.6 | 5.6 | 5.9 | 2.4 | 20.6 | 1.0 | 17.5 | 4.8 |
|  | 144 | 4.7 | 1.8 | 71.4 | 4.9 | 3.4 | 1.3 | 12.4 | 0.7 | 8.3 | 4.7 |
| **C−2028** | 24 | 2.2 | 1.3 | 61.2 | 6.7 | 7.1 | 2.0 | 20.2 | 4.2 | 9.4 | 2.8 |
|  | 72 | 26.2 | 6.1 | 40.9 | 6.3 | 5.4 | 3.4 | 22.5 | 7.2 | 5.0 | 2.5 |
|  | 144 | 39.3 | 3.2 | 35.5 | 9.4 | 4.9 | 2.8 | 14.3 | 2.9 | 6.2 | 0.5 |
| **QD_green_−C−2028** | 24 | 0.7 | 0.3 | 52.8 | 5.1 | 12.3 | 1.5 | 24.9 | 0.2 | 9.4 | 3.5 |
|  | 72 | 28.7 | 7.4 | 44.4 | 1.6 | 6.4 | 2.4 | 14.9 | 6.1 | 5.6 | 0.3 |
|  | 144 | 26.3 | 2.0 | 41.7 | 0.9 | 5.9 | 1.9 | 18.3 | 0.5 | 7.9 | 1.5 |
| **QD_green_−*β*−CD(C−2028)−FA** | 24 | 1.3 | 0.8 | 51.9 | 10.6 | 11.9 | 1.6 | 25.9 | 7.3 | 9.0 | 4.5 |
|  | 72 | 18.7 | 7.5 | 43.7 | 6.0 | 4.7 | 0.8 | 24.0 | 4.3 | 9.0 | 5.1 |
|  | 144 | 18.8 | 4.6 | 40.9 | 3.1 | 4.8 | 3.1 | 25.7 | 2.3 | 9.8 | 2.5 |

**Table S1 c.** Analysis of cell cycle by flow cytometry in LNCaP cells. Data shows the percentage of cells treated with QD_green_. QD_green_−*β*−CD−FA. C−2028. QD_green_−C−2028. and QD_green_−*β*−CD(C−2028)−FA nanoconjugates at IC_80_ value for the time indicated in the sub-G1. G1. S. G2/M. and Poli (polyploid cells) phases of the cell cycle. Data represented the averages of three independent experiments.

| **Compound** |  | **Phases of the cell cycle** | | | | | | | | | |
| --- | --- | --- | --- | --- | --- | --- | --- | --- | --- | --- | --- |
|  | **[h]** | **sub-G1** | **±** | **G1** | **±** | **S** | **±** | **G2/M** | **±** | **Poli** | **±** |
| **Control** | 24 | 3.7 | 2.3 | 70.4 | 4.6 | 7.3 | 1.9 | 15.6 | 0.7 | 2.9 | 3.4 |
|  | 72 | 5.9 | 3.0 | 70.4 | 4.1 | 4.4 | 1.0 | 16.2 | 2.9 | 3.1 | 2.0 |
|  | 144 | 4.5 | 2.6 | 83.4 | 1.5 | 2.6 | 0.8 | 7.3 | 1.0 | 2.3 | 1.8 |
| **QD_green_** | 24 | 3.0 | 2.3 | 70.7 | 1.9 | 7.5 | 2.1 | 16.0 | 0.7 | 3.5 | 2.4 |
|  | 72 | 3.2 | 0.5 | 72.5 | 8.1 | 4.6 | 0.4 | 16.6 | 4.0 | 3.1 | 3.8 |
|  | 144 | 2.9 | 1.3 | 74.3 | 5.8 | 5.0 | 1.5 | 13.1 | 2.1 | 4.7 | 1.7 |
| **QD_green_−*β*−CD−FA** | 24 | 2.2 | 1.8 | 74.4 | 0.1 | 9.2 | 0.2 | 13.7 | 1.6 | 0.6 | 0.0 |
|  | 72 | 3.9 | 1.0 | 66.0 | 4.3 | 6.5 | 2.6 | 17.7 | 2.1 | 5.9 | 4.7 |
|  | 144 | 4.5 | 3.0 | 81.8 | 3.8 | 2.7 | 1.4 | 9.2 | 3.6 | 1.7 | 1.1 |
| **C−2028** | 24 | 4.3 | 1.2 | 61.9 | 6.0 | 8.2 | 1.0 | 22.8 | 7.4 | 3.0 | 1.6 |
|  | 72 | 67.4 | 23.6 | 22.6 | 14.6 | 1.6 | 1.6 | 6.8 | 6.2 | 1.6 | 1.3 |
|  | 144 | 81.8 | 6.0 | 10.9 | 3.8 | 1.3 | 0.4 | 4.9 | 1.7 | 1.1 | 0.3 |
| **QD_green_−C−2028** | 24 | 7.5 | 3.3 | 52.9 | 7.1 | 9.4 | 4.6 | 23.0 | 5.6 | 7.2 | 6.1 |
|  | 72 | 30.0 | 1.7 | 34.5 | 7.8 | 3.0 | 0.9 | 27.0 | 5.1 | 5.5 | 3.9 |
|  | 144 | 75.8 | 13.5 | 13.0 | 7.1 | 2.3 | 1.7 | 7.0 | 3.6 | 2.0 | 1.1 |
| **QD_green_−*β*−CD(C−2028)−FA** | 24 | 10.7 | 4.1 | 44.7 | 9.8 | 5.5 | 1.2 | 27.2 | 2.6 | 11.8 | 6.6 |
|  | 72 | 23.2 | 2.2 | 37.6 | 7.7 | 2.2 | 1.9 | 32.7 | 6.0 | 4.3 | 2.8 |
|  | 144 | 57.2 | 8.1 | 20.9 | 5.2 | 3.1 | 2.4 | 15.3 | 3.9 | 3.6 | 2.0 |

**Table S1 d.** Analysis of cell cycle by flow cytometry in MRC-5 cells. Data shows the percentage of cells treated with QD_green,_ QD_green_−*β*−CD−FA, C−2028, QD_green_−C−2028, and QD_green_−*β*−CD(C−2028)−FA nanoconjugates at IC_80_ value for the time indicated in the sub-G1, G1, S, G2/M, and Poli (polyploid cells) phases of the cell cycle. Data represented the averages of three independent experiments.

| **Compound** |  | **Phases of the cell cycle** | | | | | | | | | |
| --- | --- | --- | --- | --- | --- | --- | --- | --- | --- | --- | --- |
|  | **[h]** | **sub-G1** | **±** | **G1** | **±** | **S** | **±** | **G2/M** | **±** | **Poli** | **±** |
| **Control** | 72 | 2.7 | 1.2 | 69.3 | 4.1 | 2.5 | 1.4 | 17.0 | 2.2 | 8.5 | 1.2 |
| **QD_green_** | 72 | 2.6 | 1.5 | 82.7 | 6.5 | 1.3 | 0.9 | 9.5 | 3.5 | 4.9 | 2.1 |
| **QD_green_−*β*−CD−FA** | 72 | 3.4 | 0.2 | 71.6 | 7.1 | 4.1 | 1.3 | 14.3 | 4.2 | 6.8 | 1.5 |
| **C−2028** | 72 | 4.6 | 1.3 | 62.6 | 5.0 | 4.2 | 1.1 | 23.9 | 3.6 | 4.9 | 2.5 |
| **QD_green_−C−2028** | 72 | 3.5 | 1.2 | 68.3 | 6.7 | 2.1 | 1.3 | 20.0 | 3.0 | 6.1 | 3.5 |
| **QD_green_−*β*−CD(C−2028)−FA** | 72 | 10.2 | 4.9 | 48.8 | 5.4 | 3.8 | 0.4 | 31.1 | 2.8 | 6.3 | 2.6 |

**Table S1 e.** Analysis of cell cycle by flow cytometry in PNT1A cells. Data shows the percentage of cells treated with QD_green,_ QD_green_−*β*−CD−FA, C−2028, QD_green_−C−2028, and QD_green_−*β*−CD(C−2028)−FA nanoconjugates at IC_80_ value for the time indicated in the sub-G1, G1, S, G2/M, and Poli (polyploid cells) phases of the cell cycle. Data represented the averages of three independent experiments.

| **Compound** |  | **Phases of the cell cycle** | | | | | | | | | |
| --- | --- | --- | --- | --- | --- | --- | --- | --- | --- | --- | --- |
|  | **[h]** | **sub-G1** | **±** | **G1** | **±** | **S** | **±** | **G2/M** | **±** | **Poli** | **±** |
| **Control** | 72 | 0.9 | 0.3 | 49.5 | 6.2 | 14.1 | 1.1 | 25.2 | 3.9 | 10.2 | 2.9 |
| **QD_green_** | 72 | 1.7 | 0.8 | 46.7 | 6.3 | 9.6 | 2.9 | 30.0 | 0.4 | 12.0 | 4.0 |
| **QD_green_−*β*−CD−FA** | 72 | 1.2 | 0.5 | 49.0 | 2.8 | 9.0 | 0.5 | 29.7 | 3.3 | 11.1 | 0.3 |
| **C−2028** | 72 | 10.9 | 3.5 | 39.5 | 1.3 | 8.4 | 0.3 | 28.8 | 2.0 | 12.3 | 1.3 |
| **QD_green_−C−2028** | 72 | 8.4 | 1.4 | 40.3 | 5.9 | 6.1 | 1.5 | 33.5 | 5.0 | 11.6 | 1.1 |
| **QD_green_−*β*−CD(C−2028)−FA** | 72 | 9.5 | 4.0 | 43.5 | 3.4 | 6.5 | 2.8 | 31.5 | 3.1 | 8.9 | 0.5 |

| **A** | | | **B** | | | **C** |
| --- | --- | --- | --- | --- | --- | --- |
| **72 h** | | | | | | |
| **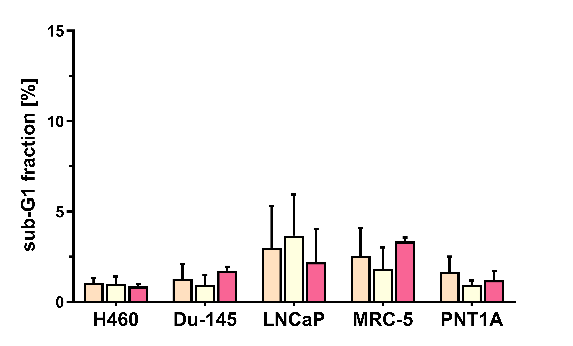** | | | **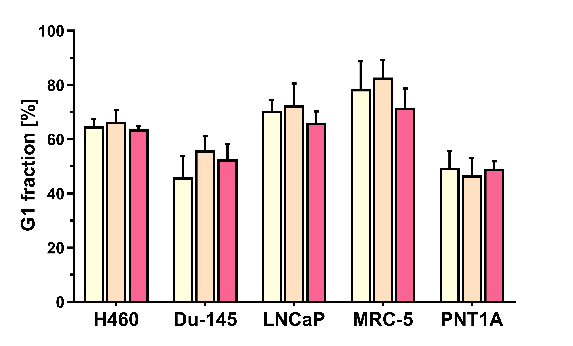** | | | **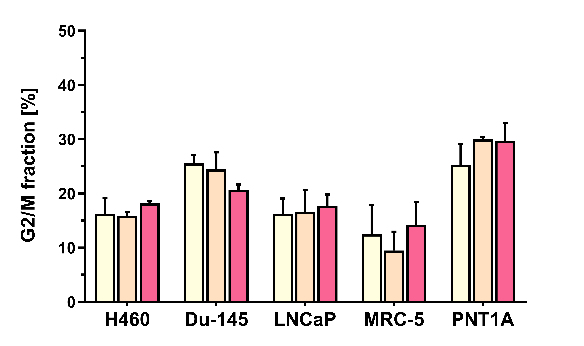** |
| **D** | **E** | | | | **F** |  |
| **144 h** | | | | | | **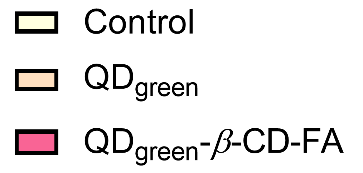** |
| **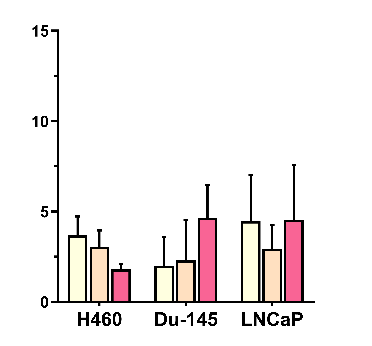** | | **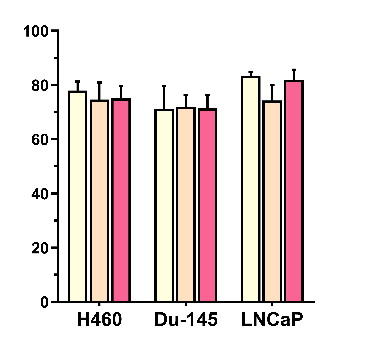** | | **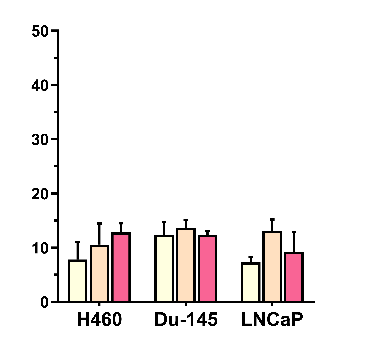** | |  |

**Figure S8.** Flow cytometry analysis of cell cycle after 72 h (**A-C**) and 144 h (**D-F**) incubation with QD_green_ and QD_green_−*β*−CD−FA in H460, Du-145, LNCaP, MRC-5, and PNT1A cells

**Table S2 a.** Flow cytometry analysis of phosphatidylserine externalization by Annexin V/propidium iodide (PI) assay in H460 cells. Percentage of cells treated with QD_green,_ QD_green_−*β*−CD−FA, C−2028, QD_green_−C−2028, and QD_green_−*β*−CD(C−2028)−FA nanoconjugates at IC_80_ value. Data represented the averages of three independent experiments. A-/PI- (Annexin V negative. PI negative) represents live cells; A+/PI- (Annexin V positive. PI negative) - early apoptotic cells; A+/PI+ (Annexin V positive. PI positive) - late apoptotic cells; A-/PI+ (Annexin V negative. PI positive) - primary necrotic cells.

| **Compound** |  | **Percentage of cell population [%]** | | | | | | | |
| --- | --- | --- | --- | --- | --- | --- | --- | --- | --- |
|  |  | **A-/PI-** | | **A+/PI-** | | **A+/PI+** | | **A-/PI+** | |
|  | **[h]** | **mean** | **SD** | **mean** | **SD** | **mean** | **SD** | **mean** | **SD** |
| **Control** | 24 | 89.9 | 3.1 | 1.6 | 1.0 | 6.2 | 2.5 | 2.1 | 1.5 |
|  | 72 | 91.9 | 1.0 | 2.4 | 1.0 | 5.0 | 1.0 | 0.7 | 0.5 |
|  | 144 | 82.5 | 6.8 | 5.9 | 4.4 | 9.7 | 3.0 | 2.0 | 1.2 |
| **QD_green_** | 24 | 92.9 | 1.9 | 2.2 | 1.1 | 4.1 | 1.3 | 0.9 | 0.7 |
|  | 72 | 91.8 | 2.0 | 2.3 | 0.7 | 5.3 | 1.8 | 0.7 | 0.5 |
|  | 144 | 89.5 | 2.9 | 2.2 | 1.1 | 7.2 | 1.9 | 1.1 | 0.6 |
| **QD_green_−*β*−CD−FA** | 24 | 92.5 | 2.0 | 1.2 | 0.6 | 5.7 | 1.3 | 0.6 | 0.4 |
|  | 72 | 92.3 | 2.0 | 1.5 | 0.8 | 5.5 | 1.9 | 0.7 | 0.6 |
|  | 144 | 91.6 | 1.6 | 0.9 | 0.7 | 6.8 | 2.1 | 0.6 | 0.4 |
| **C−2028** | 24 | 76.5 | 3.1 | 7.8 | 3.8 | 12.8 | 3.8 | 3.0 | 1.2 |
|  | 72 | 41.3 | 3.0 | 6.0 | 2.7 | 48.1 | 3.2 | 4.6 | 3.3 |
|  | 144 | 48.2 | 6.7 | 3.1 | 1.1 | 44.1 | 7.2 | 4.3 | 4.2 |
| **QD_green_−C−2028** | 24 | 86.7 | 5.1 | 3.5 | 1.2 | 8.8 | 4.8 | 1.1 | 0.6 |
|  | 72 | 76.6 | 4.4 | 4.1 | 1.5 | 16.5 | 5.4 | 2.8 | 1.1 |
|  | 144 | 69.3 | 3.0 | 2.9 | 0.9 | 23.8 | 4.2 | 4.1 | 0,6 |
| **QD_green_−*β*−CD(C−2028)−FA** | 24 | 78.1 | 1.6 | 2.8 | 2.0 | 16.2 | 1.5 | 2.8 | 2.1 |
|  | 72 | 51.3 | 2.7 | 2.7 | 2.1 | 44.4 | 1.8 | 1.7 | 1.2 |
|  | 144 | 66.2 | 2.1 | 1.5 | 1.1 | 30.4 | 2.0 | 1.9 | 0.9 |

**Table S2 b.** Flow cytometry analysis of phosphatidylserine externalization by Annexin V/propidium iodide (PI) assay in Du‑145 cells. Percentage of cells treated with QD_green,_ QD_green_−*β*−CD−FA, C−2028, QD_green_−C−2028, and QD_green_−*β*−CD(C−2028)−FA nanoconjugates at IC_80_ value. Data represented the averages of three independent experiments. A-/PI- (Annexin V negative. PI negative) represents live cells; A+/PI- (Annexin V positive. PI negative) - early apoptotic cells; A+/PI+ (Annexin V positive. PI positive) - late apoptotic cells; A-/PI+ (Annexin V negative. PI positive) - primary necrotic cells.

| **Compound** |  | **Percentage of cell population [%]** | | | | | | | |
| --- | --- | --- | --- | --- | --- | --- | --- | --- | --- |
|  |  | **A-/PI-** | | **A+/PI-** | | **A+/PI+** | | **A-/PI+** | |
|  | **[h]** | **mean** | **SD** | **mean** | **SD** | **mean** | **SD** | **mean** | **SD** |
| **Control** | 24 | 95.8 | 2.7 | 0.6 | 0.3 | 2.7 | 1.7 | 0.9 | 0.8 |
|  | 72 | 94.1 | 2.3 | 0.6 | 0.4 | 3.8 | 1.4 | 1.5 | 1.0 |
|  | 144 | 91.9 | 1.0 | 1.0 | 0.2 | 5.9 | 1.0 | 1.2 | 1.2 |
| **QD_green_** | 24 | 96.3 | 2.7 | 0.5 | 0.2 | 1.4 | 1.7 | 0.8 | 0.8 |
|  | 72 | 93.2 | 2.7 | 0.6 | 0.2 | 4.5 | 1.6 | 1.7 | 1.0 |
|  | 144 | 91.3 | 1.3 | 1.1 | 0.3 | 6.4 | 0.5 | 1.3 | 1.6 |
| **QD_green_−*β*−CD−FA** | 24 | 95.7 | 2.2 | 0.6 | 0.1 | 2.7 | 1.1 | 1.1 | 1.1 |
|  | 72 | 93.1 | 2.1 | 0.6 | 0.2 | 4.5 | 1.3 | 1.8 | 0.9 |
|  | 144 | 91.7 | 3.5 | 1.0 | 0.5 | 6.0 | 2.6 | 1.3 | 1.3 |
| **C−2028** | 24 | 91.3 | 3.6 | 0.9 | 0.2 | 5.7 | 1.6 | 2.0 | 1.0 |
|  | 72 | 51.8 | 3.6 | 1.6 | 0.7 | 44.7 | 2.6 | 1.9 | 1.4 |
|  | 144 | 25.6 | 6.1 | 4.0 | 1.8 | 69.1 | 7.8 | 1.3 | 0.1 |
| **QD_green_−C−2028** | 24 | 91.9 | 2.3 | 1.1 | 0.4 | 5.3 | 1.7 | 1.7 | 0.4 |
|  | 72 | 54.7 | 6.7 | 1.2 | 0.3 | 42.5 | 5.6 | 1.6 | 1.5 |
|  | 144 | 39.5 | 3.9 | 2.4 | 1.5 | 56.8 | 4.5 | 1.2 | 0.3 |
| **QD_green_−*β*−CD(C−2028)−FA** | 24 | 93.5 | 3.7 | 0.7 | 0.2 | 4.7 | 3.0 | 1.2 | 0.7 |
|  | 72 | 65.5 | 3.3 | 1.3 | 0.1 | 31.7 | 3.5 | 1.5 | 0.1 |
|  | 144 | 45.3 | 4.5 | 2.5 | 1.8 | 51.1 | 5.9 | 1.1 | 0.4 |

**Table S2 c.** Flow cytometry analysis of phosphatidylserine externalization by Annexin V/propidium iodide (PI) assay in LNCaP cells. Percentage of cells treated with QD_green,_ QD_green_−*β*−CD−FA, C−2028, QD_green_−C−2028, and QD_green_−*β*−CD(C−2028)−FA nanoconjugates at IC_80_ value. Data represented the averages of three independent experiments. A-/PI- (Annexin V negative. PI negative) represents live cells; A+/PI- (Annexin V positive. PI negative) - early apoptotic cells; A+/PI+ (Annexin V positive. PI positive) - late apoptotic cells; A-/PI+ (Annexin V negative. PI positive) - primary necrotic cells.

| **Compound** |  | **Percentage of cell population [%]** | | | | | | | |
| --- | --- | --- | --- | --- | --- | --- | --- | --- | --- |
|  |  | **A-/PI-** | | **A+/PI-** | | **A+/PI+** | | **A-/PI+** | |
|  | **[h]** | **mean** | **SD** | **mean** | **SD** | **mean** | **SD** | **mean** | **SD** |
| **Control** | 24 | 90.8 | 1.0 | 2.5 | 2.1 | 5.6 | 1.3 | 1.1 | 1.2 |
|  | 72 | 92.2 | 1.6 | 1.7 | 1.2 | 5.1 | 2.2 | 1.0 | 0.9 |
|  | 144 | 91.3 | 2.3 | 2.7 | 2.0 | 6.5 | 2.8 | 0.7 | 0.8 |
| **QD_green_** | 24 | 90.4 | 1.2 | 2.6 | 2.4 | 6.0 | 1.6 | 1.1 | 1.1 |
|  | 72 | 93.0 | 2.2 | 1.7 | 0.9 | 4.3 | 2.4 | 1.0 | 0.7 |
|  | 144 | 89.6 | 2.0 | 2.3 | 2.0 | 7.5 | 2.8 | 0.6 | 0.4 |
| **QD_green_−*β*−CD−FA** | 24 | 90.0 | 0.9 | 2.2 | 3.1 | 6.3 | 1.8 | 1.5 | 0.6 |
|  | 72 | 92.6 | 2.0 | 1.7 | 0.7 | 4.8 | 2.4 | 1.0 | 1.0 |
|  | 144 | 91.3 | 1.5 | 3.3 | 2.2 | 4.9 | 2.4 | 0.5 | 0.4 |
| **C−2028** | 24 | 85.4 | 4.4 | 3.8 | 2.6 | 9.2 | 4.0 | 1.6 | 0.5 |
|  | 72 | 58.5 | 13.6 | 6.2 | 0.6 | 32.8 | 9.8 | 2.8 | 3.1 |
|  | 144 | 28.6 | 9.8 | 5.5 | 2.7 | 63.4 | 9.7 | 2.5 | 1.1 |
| **QD_green_−C−2028** | 24 | 88.2 | 4.7 | 3.3 | 2.0 | 7.5 | 2.9 | 1.0 | 0.8 |
|  | 72 | 72.4 | 5.4 | 2.9 | 1.5 | 21.9 | 4.9 | 2.8 | 1.7 |
|  | 144 | 67.2 | 7.1 | 3.6 | 2.3 | 28.1 | 9.1 | 3.2 | 3.8 |
| **QD_green_−*β*−CD(C−2028)−FA** | 24 | 89.8 | 2.6 | 2.2 | 2.4 | 7.0 | 2.0 | 1.1 | 0.8 |
|  | 72 | 79.3 | 0.9 | 4.1 | 0.9 | 15.7 | 1.3 | 1.0 | 0.8 |
|  | 144 | 57.3 | 3.0 | 4.5 | 3.1 | 33.3 | 1.6 | 4.9 | 2.5 |

**Table S2 d.** Flow cytometry analysis of phosphatidylserine externalization by Annexin V/propidium iodide (PI) assay in MRC-5 cells. Percentage of cells treated with QD_green,_ QD_green_−*β*−CD−FA, C−2028, QD_green_−C−2028, and QD_green_−*β*−CD(C−2028)−FA nanoconjugates at IC_80_ value. Data represented the averages of three independent experiments. A-/PI- (Annexin V negative. PI negative) represents live cells; A+/PI- (Annexin V positive. PI negative) - early apoptotic cells; A+/PI+ (Annexin V positive. PI positive) - late apoptotic cells; A-/PI+ (Annexin V negative. PI positive) - primary necrotic cells.

| **Compound** |  | **Percentage of cell population [%]** | | | | | | | |
| --- | --- | --- | --- | --- | --- | --- | --- | --- | --- |
|  |  | **A-/PI-** | | **A+/PI-** | | **A+/PI+** | | **A-/PI+** | |
|  | **[h]** | **mean** | **SD** | **mean** | **SD** | **mean** | **SD** | **mean** | **SD** |
| **Control** | 72 | 95.4 | 2.7 | 1.7 | 1.1 | 2.5 | 1.7 | 0.4 | 0.2 |
| **QD_green_** | 72 | 96.0 | 2.8 | 1.3 | 0.4 | 2.3 | 2.3 | 0.4 | 0.4 |
| **QD_green_−*β*−CD−FA** | 72 | 95.7 | 3.1 | 1.8 | 1.7 | 2.1 | 1.4 | 0.4 | 0.3 |
| **C−2028** | 72 | 87.1 | 2.3 | 4.5 | 2.4 | 5.4 | 1.8 | 3.0 | 3.2 |
| **QD_green_−C−2028** | 72 | 89.3 | 4.8 | 5.2 | 3.0 | 4.9 | 2.8 | 0.7 | 0.8 |
| **QD_green_−*β*−CD(C−2028)−FA** | 72 | 93.7 | 1.3 | 3.7 | 1.5 | 2.3 | 0.5 | 0.4 | 0.4 |

**Table S2 e.** Flow cytometry analysis of phosphatidylserine externalization by Annexin V/propidium iodide (PI) assay in PNT1A cells. Percentage of cells treated with QD_green,_ QD_green_−*β*−CD−FA, C−2028, QD_green_−C−2028, and QD_green_−*β*−CD(C−2028)−FA nanoconjugates at IC_80_ value. Data represented the averages of three independent experiments. A-/PI- (Annexin V negative. PI negative) represents live cells; A+/PI- (Annexin V positive. PI negative) - early apoptotic cells; A+/PI+ (Annexin V positive. PI positive) - late apoptotic cells; A-/PI+ (Annexin V negative. PI positive) - primary necrotic cells.

| **Compound** |  | **Percentage of cell population [%]** | | | | | | | |
| --- | --- | --- | --- | --- | --- | --- | --- | --- | --- |
|  |  | **A-/PI-** | | **A+/PI-** | | **A+/PI+** | | **A-/PI+** | |
|  | **[h]** | **mean** | **SD** | **mean** | **SD** | **mean** | **SD** | **mean** | **SD** |
| **Control** | 72 | 93.7 | 0.9 | 1.1 | 0.5 | 4.0 | 1.3 | 1.2 | 0.5 |
| **QD_green_** | 72 | 93.0 | 1.0 | 1.1 | 0.4 | 4.5 | 0.7 | 1.5 | 0.9 |
| **QD_green_−*β*−CD−FA** | 72 | 93.3 | 0.6 | 0.9 | 0.4 | 4.3 | 0.3 | 1.4 | 0.8 |
| **C−2028** | 72 | 72.9 | 5.9 | 1.7 | 1.6 | 23.8 | 4.1 | 1.5 | 1.1 |
| **QD_green_−C−2028** | 72 | 49.3 | 0.6 | 1.7 | 1.0 | 44.9 | 3.4 | 4.1 | 2.6 |
| **QD_green_−*β*−CD(C−2028)−FA** | 72 | 44.7 | 0.3 | 1.5 | 0.7 | 50.3 | 2.7 | 3.5 | 2.2 |

| **A** | **B** | **C** |
| --- | --- | --- |
| **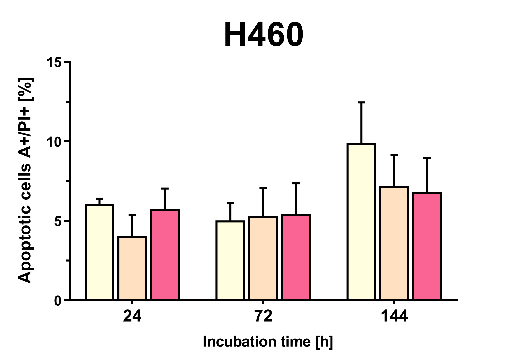** | **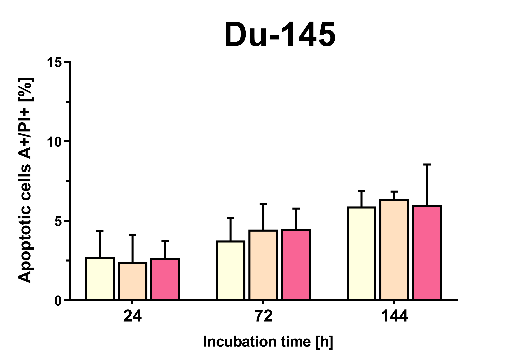** | **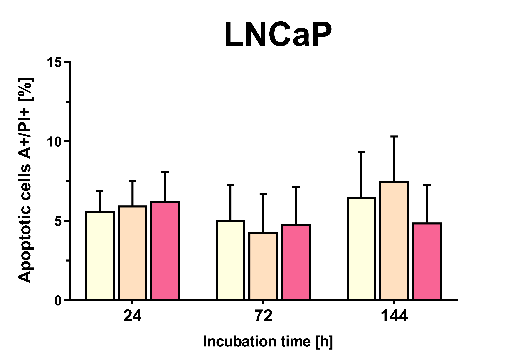** |
| **D** | **E** |  |
| **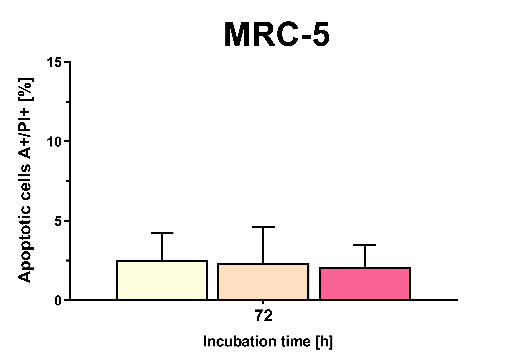** | **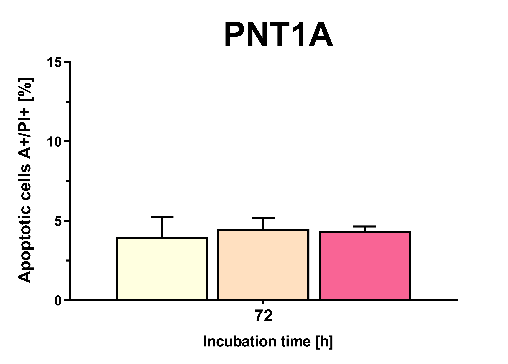** | **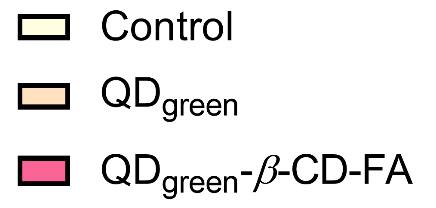** |

**Figure S9.** Flow cytometry analysis of phosphatidylserine externalization by Annexin V/propidium iodide (PI) assay in (**A**) H460, (**B**) Du-145, (**C**) LNCaP, (**D**) MRC-5, and (**E**) PNT1A cells treated with QD_green_ and QD_green_−*β*−CD−FA expressed as the percentage of late apoptotic cells (A+/PI+).


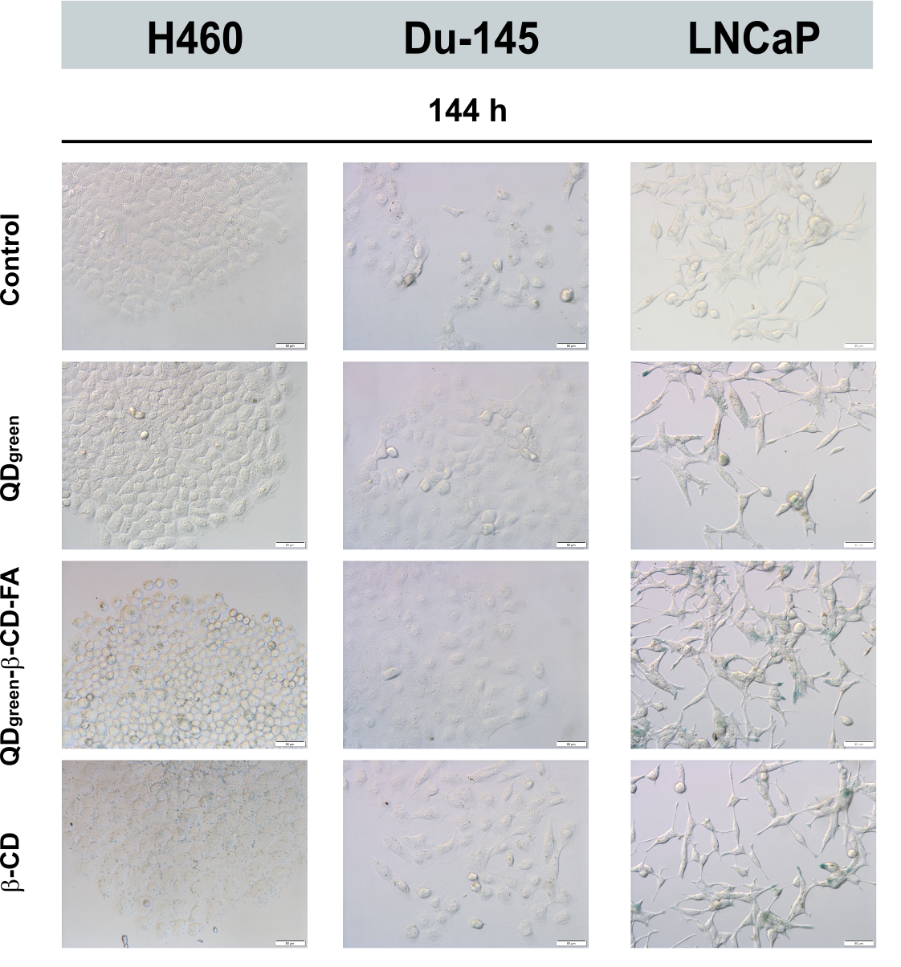


**Figure S10.** Cellular senescence of H460, Du-145, and LNCaP cancer cells following treatment with QD_green_. QD_green_−*β*−CD−FA, and *β*−CD for 144 h. Senescence-associated *β*-galactosidase activities were assessed by X-gal staining using a light microscope. Data represented the averages of three independent experiments. The scale bar is 50 µm.
